# Follicular Helper T-Cell-Based Classification of Endometrial Cancer Promotes Precise Checkpoint Immunotherapy and Provides Prognostic Stratification

**DOI:** 10.3389/fimmu.2021.788959

**Published:** 2022-01-07

**Authors:** Yi Chen, Shuwen You, Jie Li, Yifan Zhang, Georgia Kokaraki, Elisabeth Epstein, Joseph Carlson, Wen-Kuan Huang, Felix Haglund

**Affiliations:** ^1^ Department of Oncology and Pathology, Karolinska Institutet, Stockholm, Sweden; ^2^ Department of Clinical Pathology and Cytology, Karolinska University Hospital Solna, Stockholm, Sweden; ^3^ Department of Gynecology Oncology, Women’s Hospital of Zhejiang University, Hangzhou, China; ^4^ College of First Clinical Medicine, Shandong University of Traditional Chinese Medicine, Jinan, China; ^5^ Department of Clinical Science and Education, Karolinska Institutet, Stockholm, Sweden; ^6^ Department of Obstetrics and Gynaecology, Södersjukhuset, Stockholm, Sweden; ^7^ Keck School of Medicine of University of Southern California, Los Angeles, CA, United States; ^8^ Division of Hematology-Oncology, Department of Internal Medicine, Chang Gung Memorial Hospital at Linkou, Chang Gung University College of Medicine, Taoyuan, Taiwan

**Keywords:** genital neoplasms, female, immunotherapy, tumor microenvironment, tumor biomarkers, computational biology

## Abstract

Despite the fact that management of EC is moving towards four TCGA-based molecular classifications, a pronounced variation in immune response among these molecular subtypes limits its clinical use. We aimed to investigate the determinant biomarker of ICI response in endometrial cancer (EC). We characterized transcriptome signatures associated with tumor immune microenvironment in EC. Two immune infiltration signatures were identified from the TCGA database (*n* = 520). The high- and low-infiltration clusters were compared for differences in patient clinical characteristics, genomic features, and immune cell transcription signatures for ICI prediction. A Lasso Cox regression model was applied to construct a prognostic gene signature. Time-dependent receiver operating characteristic curve, Kaplan–Meier curve, nomogram, and decision curve analyses were used to assess the prediction capacity. The efficacy of potential biomarker was validated by the Karolinska endometrial cancer cohort (*n* = 260). Immune signature profiling suggested that T follicular helper–like cells (Tfh) may be an important and favorable factor for EC; high Tfh infiltration showed potential for clinical use in the anti-PD-1 treatment. A Tfh Infiltration Risk Model (TIRM) established using eight genes was validated, and it outperformed the Immune Infiltration Risk Model. The TIRM had a stable prognostic value in combination with clinical risk factors and could be considered as a valuable tool in a clinical prediction model. We identified CRABP1 as an individual poor prognostic factor in EC. The Tfh-based classification distinguishes immune characteristics and predicts ICI efficacy. A nomogram based on Tfh-related risk score accurately predicted the prognosis of patients with EC, demonstrating superior performance to TCGA-based classification.

## Introduction

Although a majority of patients with endometrial cancer (EC) are diagnosed at an early stage with a favorable 5-year survival rate of 82%, patients with advanced disease have a poor prognosis with a 5-year survival rate of approximately 20% (1). The current standard first-line treatment is the combination of platinum and paclitaxel; however, optimal treatment for the second-line setting is limited (2). Therefore, the management of advanced or recurrent EC remains challenging. Immune checkpoint blockade with anti-programmed cell death (PD)-1 and anti-PD ligand-1 (PD-L1) monoclonal antibodies has emerged as a promising treatment strategy for several cancer types, including EC (3).

Several studies have supported the definite role of immune checkpoint inhibitors (ICIs) against PD-1 in the management of EC. The KEYNOTE-158 phase II trial with pembrolizumab monotherapy reported that 49 EC patients with mismatch repair deficiency (dMMR) or high microsatellite instability (MSI-H) had an objective response rate (ORR) of 57.1%, including 8 patients showing complete response (4). A phase II trial (KEYNOTE-146) evaluated the combination of pembrolizumab and lenvatinib (a kinase targeting VEGFR1-3) in 108 patients with previously treated advanced EC (5). Among the overall population with an ORR of 38% at 24 weeks, patients with MSI-H tumors had a high ORR of 63.6%. Recently, a phase I/II GARNET trial evaluated the safety and efficacy of dostarlimab following the failure of platinum-based treatment in the largest cohort of advanced EC (*n* = 245) to date (6). An ORR of 42.3% was observed in the dMMR group (7). The ORR in the proficient MMR (pMMR) group was 36.2% and 13.4% in the KEYNOTE-146 and GARNET study, respectively (5, 6). These results highlight the need for more efficient biomarkers than MSI/dMMR to identify patients responsive to ICIs.

The Cancer Genomic Atlas (TCGA)-based molecular classification has established a reproducible and informative framework for survival prediction and drug development (8, 9). Two subgroups of EC, ultra-mutated POLE and MSI-H, were thought to be predictive biomarkers of ICI efficacy because of the exceptionally high mutational burden (10). However, a significant variation in the immune response was observed across and within the four molecular subtypes (11), suggesting that the molecular classification alone may be insufficient to assist patient selection for checkpoint immunotherapy. High tumor-infiltrating lymphocytes (TIL^high^) were proposed as a novel classification for identifying immunotherapy candidates (11, 12). Furthermore, a subset of EC with the microsatellite stable biomarker (MSS) showed high PD-L1 expression and CD8+ lymphocyte infiltration, and this subset can be treated with ICIs (13). Accordingly, an increased understanding of the immune landscape of tumor microenvironment can help identify patients who can benefit from immunotherapy and result in patient stratification in future clinical studies.

The present study aimed to comprehensively evaluate the tumor immune microenvironment (TIME) in EC using the TCGA cohort. We estimated the role of immunoscore (representing the infiltration of immune cells in tumor tissue) in the prediction of immune response and survival. Furthermore, we estimated the association of different immune cell types and survival and identified T follicular helper–like cells (Tfh) as the only favorable immune cell type in EC. Next, a Tfh-based classification was developed and characterized by multi-omics analysis. We validated a Tfh-related gene signature and developed a nomogram integrating the Tfh-related risk score and clinicopathological factors. CRABP1 was identified as an independent poor prognostic factor in EC that could serve as a potential therapeutic target in EC.

## Materials and Methods

### Data Source

Both clinical and gene expression data were obtained through the TCGA Uterine corpus endometrial carcinoma (UCEC) cohort from the NCI Genomic Data Commons (GDC) archive (14). We investigated transcriptional data in Fragments Per Kilobase of transcript per Million mapped reads (FPKM) values, and the gene expression units for downstream analyses were transformed with log2([FPKM] + 1). Samples without gene expression data, clinical information, or survival time (0 ≤ days) were excluded from analyses, resulting in a final sample size of 520 patients. The somatic mutation data of 520 EC patients were downloaded from the TCGA database in which the mutations had been called by VarScan2. For the copy number variation (CNV) profile, we downloaded the level 3 CNV dataset of EC patients in the SNP6.0 microarray from the TCGA database. We also employed the R package “TCGAbiolinks” (15) to retrieve the microsatellite instability (MSI) status (MSI-H, intermediate, MSI-L, and MSS) of the patients from the GDC data portal. We obtained an Immune-related gene set from the Tumor IMmune Estimation Resource (TIMER) database for subsequent analyses (**Table S1**) (16, 17). Immune-related signatures, including the interferon gamma (IFNγ) gene, cytolytic immune activity (CYT), and immune checkpoints, were obtained from previous publications (**Table S2**) (18–20).

### Immune-Related Scores and Clustering

The fraction of stromal and immune cells was inferred with the Estimation of STromal and Immune cells in MAlignant Tumors using Expression data (ESTIMATE) algorithm (21). Immune and stromal cells were then classified into high- and low-score groups according to the median value of immune and stromal scores. To further explore the association between these scores and EC, relevant analysis correlated with overall survival (OS) and clinical grade, and the stage of patients were performed. Subsequently, we estimated the abundance of immune signatures in each sample and quantified the immune infiltration degree by a single-sample gene set enrichment analysis (ssGSEA) algorithm, which was performed in R package GSVA (22). The 30 immune signatures, including TIME cell subsets and checkpoints from previous studies (23, 24), are shown in **Table S3**. The R package “ConsensusClusterPlus” (25) was used to construct a consistency matrix to classify the samples by clustering to generate subtypes. The “K-means” algorithm with Euclidean distance was used to perform clustering analysis with 500 iterations.

### Genomic Data Analysis

Tumor mutational burden (TMB) was defined as the total number of unique genes per Mb with non-synonymous somatic mutations in each sample. For TMB features, after merging the MAF data of TCGA-UCEC, we extracted 5,000 most frequently germline mutated genes, and the identification of significantly mutated genes was obtained from robust driver gene studies (26). The TMB score formula for each sample was calculated as follows (27):

Total number of truncating mutations*1.5 + Total number of non-truncating mutations*1.0.

Truncating mutation category contains frame-shift deletion or insertion, nonsense, and splice-site mutations, while non-truncating mutation category contains in-frame deletion or insertion, missense, and nonstop mutations (**Table S4**). We analyzed the CNV profile with GISTIC2.0 software (28) and the parameters are set as follows:

*-ta 0.1 -armpeel 1 -brlen 0.7 -cap 1.5 -conf 0.9 -td 0.1 -genegistic 1 -gcm extreme -js 4 -maxseg 2000 -qvt 0.25 -rx 0 -savegene 1.*

The SNP and CNV data were then analyzed and visualized using the “oncplot” function with the R package “maftools” (29). According to one of the most influential molecular classification of EC proposed by TCGA (8), 520 patients were grouped into four subtypes: POLE (ultramutated), MSI (hypermutated), copy-number low (endometrioid), and copy-number high (serous-like).

### Development of the Prognostic Gene Expression Signature

We divided 60% of the 520 EC samples randomly into a training group to construct a prognostic signature. The remaining 40% of samples were defined as a validation group for accessing to the prognostic signature performance. There were no statistically significant differences in any clinical feature between the training group and validation group, indicating that the samples were successfully split randomly (**Table S5**).

After correlating the expression levels of the DEGs with the survival time, a univariate Cox proportional regression analysis was performed to screen for DEGs related to survival. The significant survival-related gene set was subjected to penalized multivariate Cox proportional hazards survival modeling by an algorithm for variable selection based on L1-penalized Lasso (L1-Least Absolute Shrinkage and Selection Operator) estimation (30). The construction of the survival modeling process was repeated 1,000 times, and the resulting models were subsequently combined through cross-validation during these iterations. Consequently, a risk model was constructed and the risk score formula for each patient was established by comparing each of the selected genes by their estimated regression coefficients from the Lasso regression analysis as discussed in previous studies (31, 32). Patients were separated into high- and low-risk groups according to the median value of risk score. A time-dependent receiver operating characteristic (ROC) curve analysis was conducted to measure the prognostic performance.

### Differences Between High- and Low-Infiltration Groups

Several differences in various characteristics between the high- and low-infiltration groups were carefully studied. First, clinical characteristics such as age, BMI, race, histological type, clinical stage, grade, survival status, and TCGA classification of the two groups were compared through the Chi-square or Fisher exact test. The prognosis, immune cell type, and functional enrichment between the two groups were performed and analyzed. Then, the differential gene expression analysis of the two groups was performed through the R package “limma” at the cutoff of FDR < 0.05 and absolute log2FC > 0.3. The status of clustered datasets was plotted using the “ComplexHeatmap” (33) R package. In order to explore the significantly enriched pathways and view the function of these differentially expressed genes (DEGs), Gene Ontology (GO) biological processes terms and Reactome pathway analysis were performed by Gene Set Enrichment Analysis (GSEA 4.1.0) (34, 35) or implemented by (36) Metascape and simplifyEnrichment package in R (37). Additionally, by taking the intersection of DEGs with the immune-related gene set, the DEGs related to immunity were obtained.

### High and Low Significant Infiltration Group in Specific Immune Cell

The hierarchical clustering algorithm clustered a total of 30 immune cells. The connection relationship among these cells was established through correlation, and their relationship with OS was calculated. Next, the univariate Cox proportional regression analysis was performed to identify immune cells that are significantly associated with prognosis. Subsequently, the dichotomization threshold of the significant immune cell infiltration within the high and low groups was determined by the median value of the significant immune cell infiltration. By performing differential expression analysis on the two groups, DEGs related to immunity were finally identified. Based on this information, a specific immune cell Infiltration Risk Model was constructed for prognosis and survival.

### Prognostic Evaluation of Risk Model

To assess the prognostic risk factors, we established prognostic nomograms based on the specific immune cell Infiltration Risk Model to predict the 1-, 3-, or 5-year OS of EC. Nomograms incorporating the risk model, age, histological type, stage, and grade were constructed to predict OS based on the Cox proportional hazards model. To confirm the clinical benefits associated with the use of our nomogram, decision curve analysis (DCA) was performed.

### Immunohistochemistry

In order to investigate the prognostic value of CRABP1 immunohistochemistry, a previously characterized cohort of 266 patients with endometrial cancer was investigated (38). In short, all patients were operated at the Karolinska University Hospital between 2012 and 2015. Formalin-fixed paraffin embedded (FFPE) material were identified through the archives of the Department of Clinical Pathology and Cytology at Karolinska University Hospital. Clinical data were retrieved from digital patient records and were available for all patients. As previously described, a tissue microarray (TMA) was constructed.

The TMAs were stained for CRABP1 using a monoclonal mouse antibody (Clone C-1 from ThermoFisher Scientific, Waltham, CA, USA). The antibody dilution was evaluated on anonymized cases of breast cancer and endometrial cancer, and very limited variation in the immunoreactivity was seen across the tumor tissue. After reviewing the controls, an antibody dilution of 1:1,000 was used. Antigen retrieval was performed for 20 min in 95°C citrate-based buffer. The primary antibody was incubated overnight in 4°C and visualized using the VECTASTAIN Elite ABC-HRP Kit (Vector Laboratories, Burlingame, CA). After an initial review of the cases by two clinical pathologists, the staining intensity was arbitrarily categorized as 0 = none, 1 = weak, 2 = moderate, 3 = strong and determined by consensus. The majority of staining was cytoplasmic, but in cases with stronger immunoreactivity, nuclear staining could be seen in a subgroup of the tumor cells.

### Other Immunotherapy Cohorts Used in This Study

For the training cohort, transcriptomic and corresponding clinical data of 47 patients diagnosed with metastatic melanoma and treated with anti-CTLA-4 (cytotoxic T-lymphocyte- associated protein 4) or anti-PD-1 (programmed cell death protein 1) blockade were obtained (39). We inferred the possibility of anti-CTLA-4 and anti-PD-1 response immunotherapy in the subgroups using the “Submap” algorithm (40). We used the “IMvigor210CoreBiologies” package to retrieve 348 transcriptomic and corresponding clinical data from the IMvigor210 cohort with metastatic urothelial cancer treated with an anti-PD-L1 agent (Atezolizumab) (41). Additionally, 101 transcriptomic and corresponding clinical data of patients diagnosed with metastatic melanoma and treated with anti-PD-1 agent (Nivolumab) were used as a validation cohort (16).

### Statistical Analysis

Student’s *t*-tests were used for comparisons between two continuous and normally distributed variables. Variables that were not normally distributed were analyzed by Mann–Whitney *U* tests or Wilcoxon rank-sum tests. For comparison of three groups or more, one-way analysis of variance (ANOVA) and Kruskal–Wallis test were performed for parametric and non-parametric methods, respectively (42). Log-rank test Kaplan–Meier curve and Cox regression for survival analyses were conducted using the R packages “survminer” and “survival.” Additionally, R package “glmnet” was used to perform L1-penalized Lasso regression, ROC curve, nomogram, and DCA analyses using the R packages “pec,” “pROC” and “pROC”, “regplot”, and R function “stdca.R,” respectively (43–45). R version 4.0.3 (R Foundation for Statistical Computing, Vienna, Austria) was used to execute all statistical tests and plots.

## Results

### Construction of an Overall Immune Signature

To predict the infiltration of non-tumor cells, the immune and stromal scores were calculated by analyzing gene expression signatures associated with immune and stromal cells. To associate the immune and stromal scores with patient survival and tumor grade, we classified the 520 EC samples into upper and lower halves (high and low) based on their median scores. The Kaplan–Meier survival curves (**Figure S1A**) showed a significant difference in OS between immune score high and low groups, but no significant difference was observed when compared to the stromal score or tumor purity score (**Figures S1B, C**). The immune scores decreased with increasing grade as expected, whereas no difference was observed in relation to stage (**Figures S1D, E**).

### Development of an Immune Infiltration Risk Model

**Figure 1:**
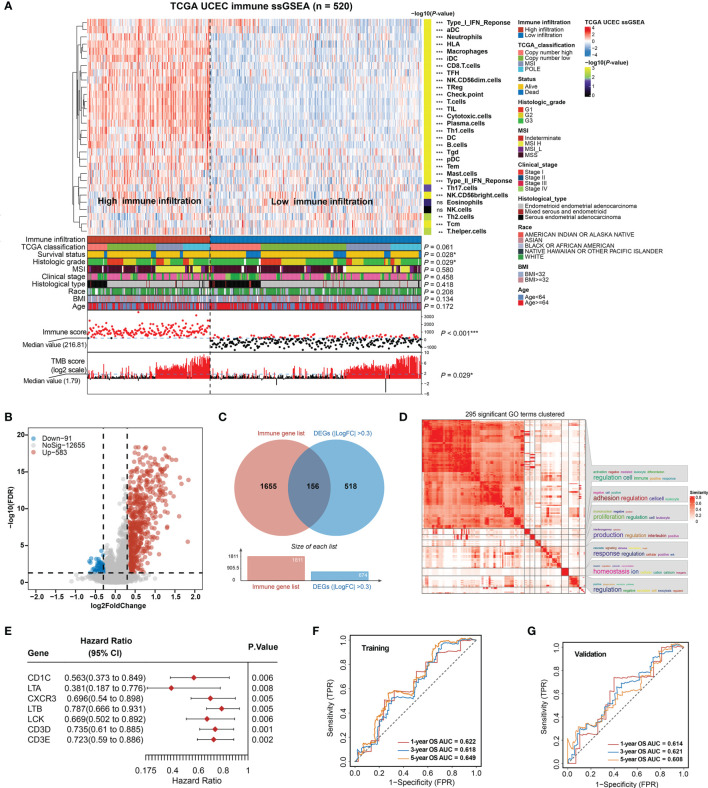
Construction of total immune signatures and Immune Infiltration Risk Model (IIRM). **(A)** SsGSEA results of 520 EC specimens, organized by total immune cell infiltration levels and immune scores. Unsupervised consensus clustering grouped gene sets into two major categories based on total immune cell infiltrations. Clinicopathological characteristics of the 520 EC patients are shown in the annotation, and different colors represent the characteristics and subtypes. The statistical differences in variables between two clusters were compared using the Fisher’s exact test. *p < 0.05, **p < 0.01, ***p < 0.001. **(B)** Volcano plots of the distribution of the DEGs between high- and low-immune infiltration groups. Red and blue represent upregulated and downregulated genes, respectively. Genes with |log2FC| > 0.3 are annotated in the plot. **(C)** Venn diagram depicts the overlap of the genes involved in immune genes and DEGs of total immune infiltration. **(D)** The 295 significant GO terms clustering in the DEGs between high- and low-immune infiltration groups. There are word cloud annotations on the right side of the heatmap that summarize the features with keywords in each GO cluster. The color shade reflects the similarity of the pathway enrichment, while different dimensions represent the size of the enrichment. **(E)** Forest plot of the univariate analyses for seven significant immune-related DEGs of total immune infiltration with overall survival (*p* < 0.01). **(F)** ROC curve of the prognostic values of IIRM training group in 1-, 3-, and 5-year OS with AUC = 0.622, 0.618, and 0.649, respectively. **(G)** ROC curve of the prognostic values of IIRM validation group in 1-, 3-, and 5-year OS with AUC = 0.614, 0.621, and 0.608, respectively. ns, non significant.

We applied ssGSEA analysis of the 520 EC tumors to quantify the signatures associated with the activity of different immune cell types. Based on 30 ssGSEA scores in each sample (**Table S6**), unsupervised clustering of the EC immune cell signatures clearly divided the 520 tumors into two groups with clustering stability decreasing for *k* = 2–10 (**Figure 1A**; **Figure S2**). As a result, a total of 191 patients were classified in the high infiltration group and 347 in the low infiltration group.

To determine whether there is any significant clinical difference between high- and low-immune infiltration tumors, we compared the clinical and genomic characteristics of the groups. As expected, the high-immune infiltration group had lower tumor purity (**Figure S3A**) and better OS (log-rank test, *p* = 0.019) (**Figure S3B**). We observed that the low-immune infiltration group contained more death cases and was significantly associated with survival status (Fisher’s exact test*, p* = 0.028), high grade (Fisher’s exact test*, p* = 0.029), low TMB (Fisher’s exact test*, p* = 0.029), and low immune score (Fisher’s exact test*, p* < 0.001), but it was not associated with TCGA classification, MSI, stage, histological type, race, BMI, and age (Fisher’s exact test*, p* > 0.05) (**Figure 1A**), while in the high-immune infiltration group, ssGSEA analysis of the immune cell composition showed that most immune cell types were more prevalent (*p* < 0.05), with the exception of Tcm and Th2 cells (enriched in the low-immune infiltration group), but no significant difference of eosinophils and NK cells. Unlike the low-immune infiltration group, the patients exhibited favorable prognosis, low grade, and high TMB (**Figure 1A**). The total immune infiltration grouping reflects some molecular and genomic characteristics of the tumor in a certain extent, regardless of the flaws in some clinical features. Since previous publications have emphasized the key role of MSI, genomic alternation in EC (8, 46), our total immune grouping only demonstrates partial significance, so it is essential to test the validity of this model hereinafter.

After identifying DEGs (FDR < 0.05, |log2FC > 0.3|) between high- and low-immunity groups, a volcano plot showed that the high-immunity group was defined by differentially overexpressed genes, where 583 were significantly overexpressed, and 91 were downregulated (**Figure 1B**). Additional pathway enrichment analysis was performed for the two groups. The most enriched GO biological processes emphasized signatures related to immune activation and regulation, where lymphocyte activation and immune effector process placed the top of the enrichment (**Figure 1D** and **Figure S3C**). Therefore, we overlapped the DEGs with the immune gene list (TIMER) and obtained 156 immune-related DEGs (**Figure 1C**), which were considered to be the most critical genes involved in immune activities. As a result, seven out of 156 DEGs were identified as individually significant favorable prognostic factors using a univariate Cox proportional regression analysis (*p* < 0.01), which are CD1C, LTA, CXCR3, LTB, LCK, CD3D, and CD3E (**Figure 1E**).

Next, the EC samples were randomly divided into a training cohort and validation cohort. From 1,000 iterations of Lasso-penalized multivariate modeling, we obtained four candidate favorable genes (CD1C, LTA, LTB, and CD3D) to construct the IIRM (**Figures S4A, B**). The AUC of the IRIM in the 1-, 3-, and 5-year OS predictions was 0.622, 0.618, and 0.649 in the training cohort and 0.614, 0.621, and 0.608 in the validation cohort, respectively (**Figures 1F, G**). Patients were subsequently divided into high- and low-risk groups according to the median value of risk score. The Kaplan–Meier survival estimates showed no significant difference in the training cohort (log-rank test, *p* = 0.389) but the high-risk group had a worse prognosis in the validation cohort (log-rank test, *p* = 0.042) (**Figures S4C, D**). Collectively, our study, by clustering EC patients based on total immune infiltrations, reveals unsatisfactory risk stratification, and suggests the complexity of the role of immune cells in the EC microenvironment.

### Identification of Key Immune Cell Types and Refinement of IIRM

**Figure 2:**
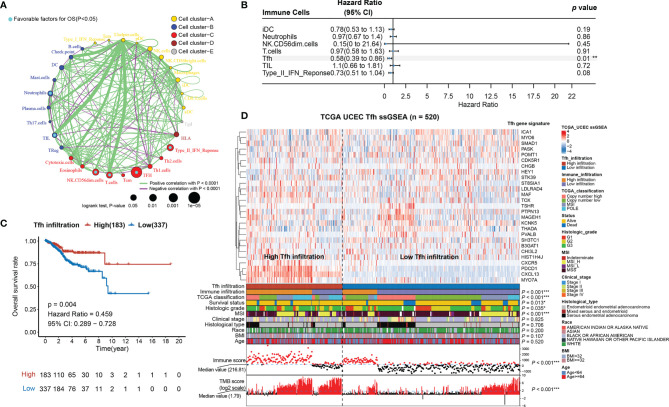
Tfh is a significant favorable cell for OS in EC. **(A)** Cellular interaction circus plot of the immune cell types. The size of each circle indicates the survival impact of each immune cell type and is inversely proportional to the *p*-value when using the log-rank test formula. Cell clusters are represented in yellow, blue, red, brown, and gray for clusters A, B, C, D, and E, respectively. The line thickness represents the estimated value of the Spearman correlation coefficient for each cell type (*p* < 0.0001). A positive and negative correlation is represented in green and purple, respectively. Favorable immune cells for overall survival are indicated in turquoise. **(B)** Forest plot of the multivariate analyses of seven significant immune cells with overall survival (**p < 0.01). **(C)** Kaplan–Meier curve of overall survival rates in EC patients with high-Tfh and low-Tfh signature (*p* = 0.004). **(D)** Tfh gene signatures of 520 EC specimens, organized by Tfh infiltration levels. Unsupervised consensus clustering grouped gene sets into two major categories based on Tfh infiltrations. Clinicopathological characteristics of the 520 EC patients are shown in the annotation, and different colors represent the characteristics and subtypes. The statistical differences in variables between two clusters were compared using the Fisher’s exact test. *p < 0.05, **p < 0.01, ***p < 0.001.

Since the AUC of IIRM did not perform very well, we next investigated the correlation between the 30 immune cell signatures (used in the hierarchical clustering algorithm) and patient survival. The results indicated that all immune signatures (including iDC, Neutrophils, NK CD56dim cells, T cells, Tfh, TIL, and Type_II_IFN_response) were significantly associated with improved outcomes (**Figure 2A**). After univariate and multivariate Cox regression analysis, Tfh was the only immune cell signature that remained significantly associated with OS (**Table S7** and **Figure 2B**). Consensus clustering analysis of the Tfh signature classified 337 tumors to the low-Tfh infiltration group while 183 belonged to the high-Tfh infiltration group with clustering stability decreasing for *k* = 2–10 (**Figure S5**). As expected, the high-Tfh infiltration group had better prognosis (log-rank test, *p* = 0.004) (**Figure 2C**).

We found that Tfh-based classification was significantly associated with immune infiltration, TCGA classification, MSI, immune score, TMB score (Fisher’s exact test*, p* < 0.001), grade (Fisher’s exact test*, p* = 0.035), and survival status (Fisher’s exact test*, p* = 0.011); however, it was not associated with stage, histological type, race, BMI, and age (Fisher’s exact test*, p* > 0.05) (**Figure 2D**; **Figure S6**). When comparing the high-Tfh infiltration group with the low-Tfh infiltration group, we found significant covariance with 23 other immune features (**Figure S7A**), similar to the pattern seen in tumors with high overall immunity (**Figure 1A**). The Tfh score showed high correlations (Spearman rank correlation, *r* = 0.62, *p* < 0.001) with the TIL score (**Figure S7B**), which is consistent with the study of Talhouk et al. demonstrating that TIL classification is a biomarker for ICIs (11).

### Genomic Variation Features of EC With Tfh-Based Classification

**Figure 3:**
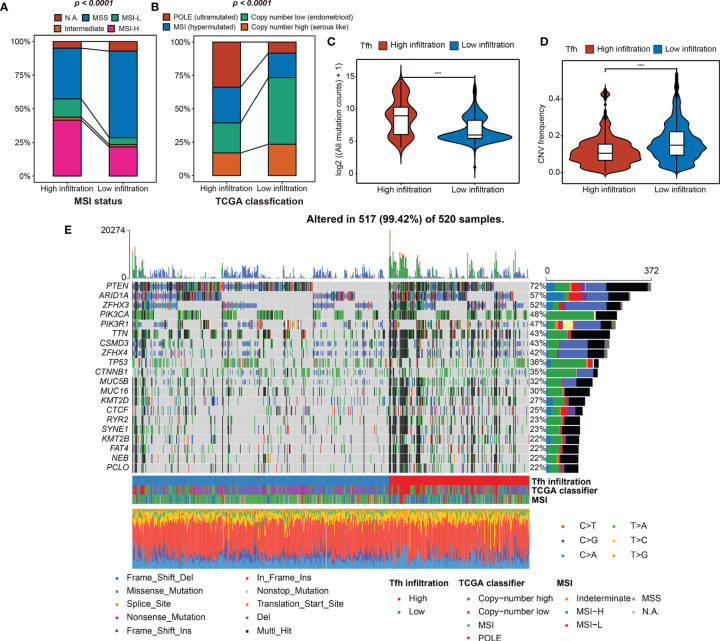
Tfh infiltration subtypes and related genomic features. **(A, B)** The fraction of MSI status, TCGA classification, shown in high- and low-Tfh infiltration groups. **(C, D)** Distribution of the mutation counts (log2 scale) **(C)** and CNV frequency **(D)** in high- and low-Tfh infiltration groups. Middle line: median; box edges: 25th and 75th percentiles, whiskers: most extreme points. ***p < 0.001, Wilcoxon rank-sum test. **(E)** The genomic profiles of 520 EC patients. Top: mutation counts of the mutated genes in each patient. Bottom: The 520 patients and immune and genomic subtypes. Right: Gene variant types and their frequencies are shown by a bar plot.

Next, the indicative genomic characteristics of the samples: MSI, TMB, CNV, and TCGA classification were used to evaluate the efficacy of Tfh clustering in EC. We can clearly observe that the high-Tfh infiltration group gathered more MSI (hypermutated) and POLE (ultra-mutated) cases than the low-Tfh infiltration group (**Figures 3A, B**), which have been proven as indicators of favorable clinical relevance (8, 47, 48).

To clarify the differences between two subgroups at the genomic level in detail, we found that the mutation events were significantly greater in the high-Tfh infiltration group, including insertion (INS), deletion (DEL), and single-nucleotide polymorphism (SNP) (Wilcoxon rank-sum test, *p* < 0.001) (**Figures S8A–C**; **Figure 3C**). In terms of CNV, the landscapes of the two subgroups are shown in **Figure S8D**. High CNV was significantly associated with low Tfh infiltration in EC and vice versa as shown in **Figure 3D** (Wilcoxon rank-sum test, *p* < 0.001). We can infer from the oncoprint plot that most of the genes had similar gene mutational and CNV profiles between the two subgroups, where PTEN was altered in 72% of all cases (**Figure 3E**). Additionally, genome instability appeared to have an apparent relationship with the level of Tfh infiltration, where more gene variations, MSI, and most specifically, POLE mutations can be found to be enriched in the high-Tfh infiltration group (**Figure 3E**), as shown in **Figures 3A, B**. From the statistical point of view of the number of mutated genes in the samples, the proportion of gene alternations in the high Tfh-infiltrated group was significantly greater than in the Tfh-infiltrated group (*P* < 0.01) (**Figure S8E**). These results suggested that Tfh infiltration may have a distinct impact on the biology of tumors in genomic features (**Figure S8F**).

### Tfh Infiltration Predicts Efficacy of Checkpoint Immunotherapy in EC

**Figure 4:**
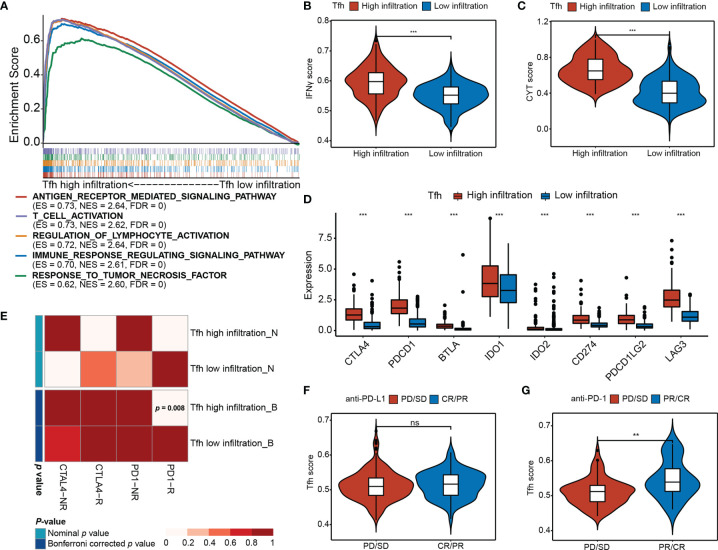
Differential immunotherapeutic response in Tfh subtypes. **(A)** GSEA of the top five significant Gene Ontology biological processes based on the enrichment scores. FDR < 0.01. **(B, C)** Distribution of the IFNγ score **(B)** and CYT score **(C)** in high- and low-Tfh infiltration groups. Middle line: median; box edges: 25th and 75th percentiles, whiskers: most extreme points. ***p < 0.001, Wilcoxon rank-sum test. **(D)** Distribution of the gene expressions (*y*-axis) of eight immune inhibitory checkpoints in high- and low-Tfh infiltration groups. Middle line: median; box edges: 25th and 75th percentiles, whiskers: most extreme points. ***p < 0.001, Wilcoxon rank-sum test. **(E)** Submap analysis of the published dataset with immunotherapy response data indicates that the Tfh high infiltration group could be more sensitive to the anti‐PD‐1 therapy (Bonferroni‐corrected *p* = 0.008). **(F, G)** Distribution of the Tfh score of anti-PD-L1 therapy status **(F)** and anti-PD-L1 therapy status **(G)** in high- and low-Tfh infiltration groups. Middle line: median; box edges: 25th and 75th percentiles, whiskers: most extreme points. **p < 0.01, Wilcoxon rank-sum test. ns, non significant.

To investigate the differences in biological processes between the two subgroups, gene set enrichment analysis (GSEA) revealed that high Tfh infiltration was mainly associated with antigen receptor-mediated signaling pathway (ES = 0.73, NES = 2.64, FDR = 0), T-cell activation (ES = 0.73, NES = 2.62, FDR = 0), regulation of lymphocyte activation (ES = 0.72, NES = 2.64, FDR = 0), immune response regulating signaling pathway (ES = 0.70, NES = 2.61, FDR = 0), and response to tumor necrosis factor (ES = 0.62, NES = 2.60, FDR = 0) (**Figure 4A**), indicating that Tfh may represent important players to be considered for linking highly efficient checkpoint immunotherapy in EC. Consistently, we found that IFNγ, which is predominantly produced by T helper (TH) CD4 and CD8 cytotoxic T lymphocyte (CTL) effector T cells during antigen-specific immunity, was significantly upregulated in the high-Tfh infiltration group (Wilcoxon rank-sum test, *p* < 0.001) (**Figure 4B**). Additionally, cytolytic immune activity (CYT) was significantly positively associated with Tfh (Wilcoxon rank-sum test, *p* < 0.001) (**Figure 4C**); it appears that a high mutation burden may generate abundant neoantigens, leading to enhanced antitumor immunity and a high CYT score in EC.

Additionally, the expression of most chemokines and chemokine receptors in the high-Tfh infiltration group was significantly greater than that in the low-Tfh infiltration group (**Figures S9A, B**) (Wilcoxon rank-sum test, *p* < 0.05), suggesting that the diverse Tfh infiltration may result in differences in immunotherapy effects to a certain extent. Next, we estimated the expression levels of eight critical inhibitory immune checkpoints, namely, CTLA4, PDCD1 (PD-L1), BTLA, IDO1, IDO2, CD274 (PD-1), PDCD1LG2 (PD-L2), and LAG3. As expected, inhibitory immune checkpoints were overexpressed in the high-Tfh infiltration group (Wilcoxon rank-sum test, *p* < 0.001) (**Figure 4D**). The Tfh scores show strong correlations with the expression of these checkpoints (i.e., CTLA4, PD-1 and PD-L1, Spearman rank correlation, *p* < 0.001, and *r* = 0.57, 0.33, and 0.62, respectively) (**Figures S10A–D**). Hence, we address the thought that targeting CTLA-4, PD-1, or PD-L1 will critically influence the function of Tfh cells in patients that receive these checkpoint immunotherapies.

We further performed a subclass mapping approach to compare the expression profile of the two Tfh subtypes with another published dataset involving 47 melanoma patients treated with CTLA-4 and PD-1 (39). We were able to observe that the high-Tfh infiltration group was more likely to be responsive to anti-PD-1 treatment than the low-Tfh infiltration group (Bonferroni corrected, *p <*0.008); however, there was no significant difference in response to the anti-CTLA-4 treatment (**Figure 4E**). Additionally, to investigate the response to anti-PD-L1 therapy, we used a cohort (IMvigor210) of 348 patients diagnosed with metastatic urothelial cancer who underwent Atezolizumab treatment to compare the Tfh score (41). We found that the complete response (CR)/partial response (PR) patients had a higher Tfh score than the progressive disease (PD)/stable disease (SD) patients, but these differences were not statistically significant (Wilcoxon rank-sum test, *p* > 0.05) (**Figure 4F**). After ssGSEA analysis of Tfh signatures, the proportion of CR/PR in the high infiltration group was a bit higher than that in the low infiltration group (Fisher’s exact test*, p* = 0.257) (**Figure S10E**). As a validation cohort (16), 101 metastatic melanoma patients treated with anti-PD-1 agent (Nivolumab) were examined to verify the favorable response to anti-PD-1 immunotherapy in the high-Tfh infiltration group. Consequently, the high Tfh score predicted a desirable clinical response to anti-PD-1 blockade (Wilcoxon rank-sum test, *p* < 0.01) (**Figure 4G**), and the proportion of CR/PR in the high infiltration group was significantly higher than that in the low infiltration group (Fisher’s exact test, *p* < 0.001) (**Figure S10F**). Thus, we considered the potential clinical usefulness of Tfh stratification in the anti-PD-1 treatment in EC.

### Evaluation of the Prognosis and Clinical Benefits of TIRM

**Figure 5:**
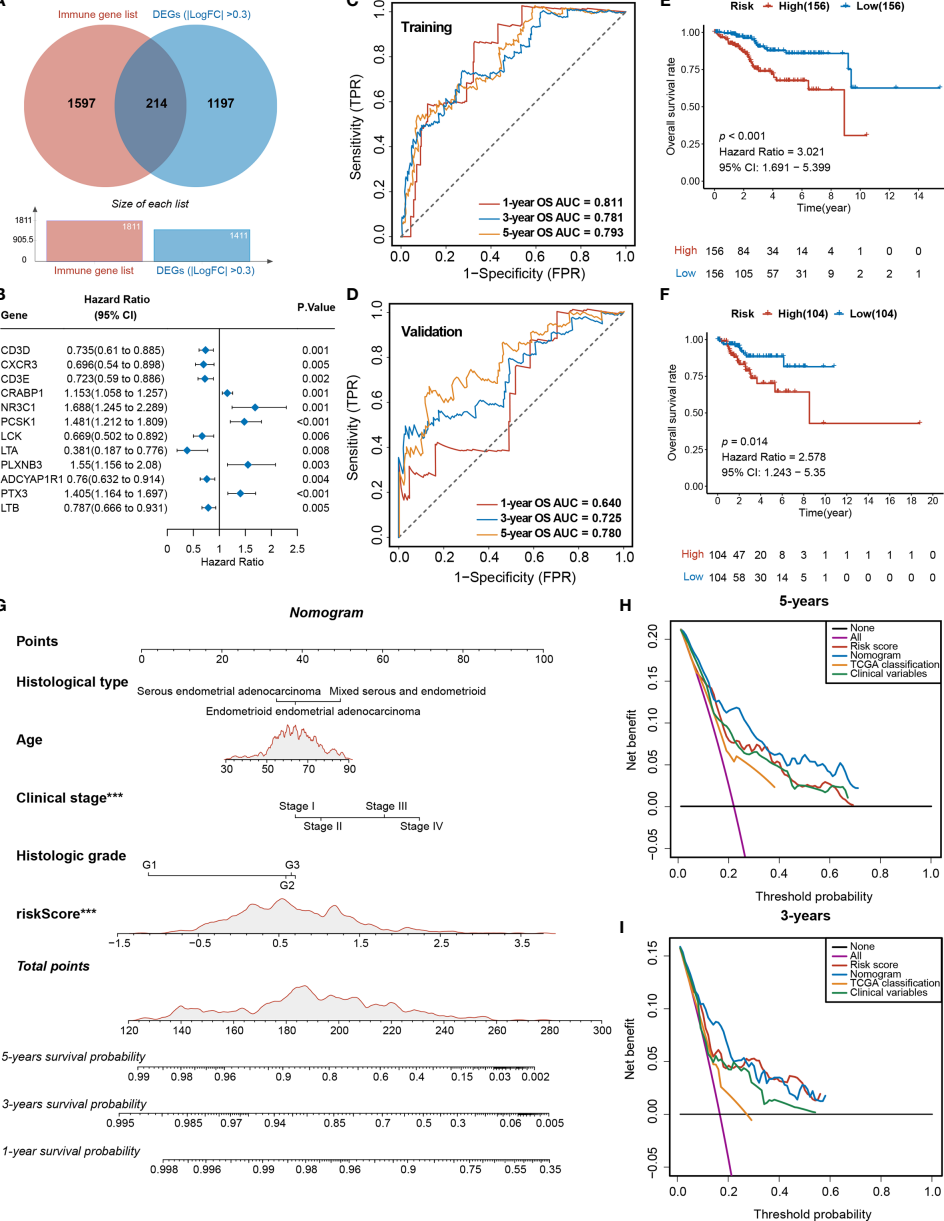
Analyses of the DEGs and development of the TIRM based on immune-related DEGs of Tfh infiltration. **(A)** Venn diagram depicts the overlap of the genes involved in immune genes and DEGs of Tfh infiltration. **(B)** Forest plot of the univariate analyses for seven significant immune-related DEGs of Tfh infiltration with overall survival (*p* < 0.01). **(C)** ROC curve of the prognostic values of TIRM training group in 1-, 3-, and 5-year OS with AUC = 0.811, 0.781, and 0.793, respectively. **(D)** ROC curve of the prognostic values of TIRM validation group in 1-, 3-, and 5-year OS with AUC = 0.640, 0.725, and 0.780, respectively. **(E, F)** Kaplan–Meier curve of the overall survival for patients in the Tfh-based high-risk and low-risk subgroups in the training group **(E)** and validation group **(F)** (*p* < 0.001 and *p* = 0.014, respectively). **(G)** A newly constructed nomogram including clinical characteristics of age, histological type, stage, grade, and risk score of Tfh in the nomogram. Each of these factors generates points according to the axes drawn upward. The total score of these components for each patient is located on the “total score” axis, which corresponds to the 1-, 3-, and 5-year OS probabilities plotted on the lower two axes. ***p < 0.001. **(H, I)** Net DCA curves show the net benefits of the nomogram at 5-year **(H)** and 3-year **(I)** OS, and the *y*-axis measures the net benefit. The nomogram has a higher net benefit compared to risk score, TCGA classification, and combined (histological type + stage + grade) and simple strategies such as follow-up of all patients (purple line) or no patients (horizontal black line) across the full range of threshold probabilities.

To further explore the differences in gene expression levels between the two subgroups, a total of 561 downregulated DEGs and 850 upregulated were identified (FDR < 0.05, |log2FC > 0.3|) (**Figure S11A**), where 241 genes were immune related (**Figure 5A**). A total of 12 of these genes were significantly associated with survival (*p* < 0.01) (**Figure 5B**). After 1,000 iterations of a Lasso-penalized multivariate model, an eight-gene-based risk model called Tfh Infiltration Risk Model (TIRM) was built, including LTA, CRABP1, PTX3, PCSK1, PLXNB3, LTB, ADCYAP1R1, and NR3C1 (**Figures S11B, C**). The ROC curve indicated better prognostic performances of the AUC (1-, 3-, and 5-year OS predictions were 0.811, 0.781 and 0.793 in the training cohort and 0.640, 0.725, and 0.780 in the validation cohort, respectively; **Figures 5C, D**) than the IIRM. The samples in training and validation cohorts were subsequently separated into high- and low-risk groups according to the median risk scores. Assessments of the Kaplan–Meier estimates showed that high-risk patients had a significantly worse OS than the low-risk patients in both cohorts (log-rank test, *p* < 0.001 and *p* = 0.014, respectively) (**Figures 5E, F**). Next, we projected this risk model to all 520 EC patients; the AUC values were all greater than 70% (1-, 3-, and 5-year OS predictions were 0.741, 0.759, and 0.786, respectively) (**Figure S11D**) and high-risk score patients had the worse prognosis (log-rank test, *p* < 0.001) (**Figure S11E**), suggesting a promising prognostic predictive ability in EC.

The indicative clinical characteristics of the samples, including the age, histological type, stage, and grade were used to evaluate the efficiency and stability of TIRM. Nomograms combining these variables as well as Tfh risk scores were generated, then the 1-, 3-, and 5-year survival probabilities were projected to the final sum of the scores (**Figure 5G**). The calibration plots of the nomogram agreed with the predictions of 1-, 3-year, and 5-year OS (**Figures S11F–H**). Subsequently, DCA for the nomogram and Tfh risk score prediction model is shown in **Figure 5H**; it can be observed that our risk model and nomogram (age + histological type + stage + grade + Tfh risk score) performed better than clinical variables (histological type + stage + grade) and TCGA classification in both 3- and 5-year OS (**Figures 5H, I**). In 1-year OS prediction, the net benefits were not calculable in independent variables, including clinical variables and TCGA classification (**Figure S11I**).

### Identification of Prognostic Biomarkers Based on TIRM

**Figure 6:**
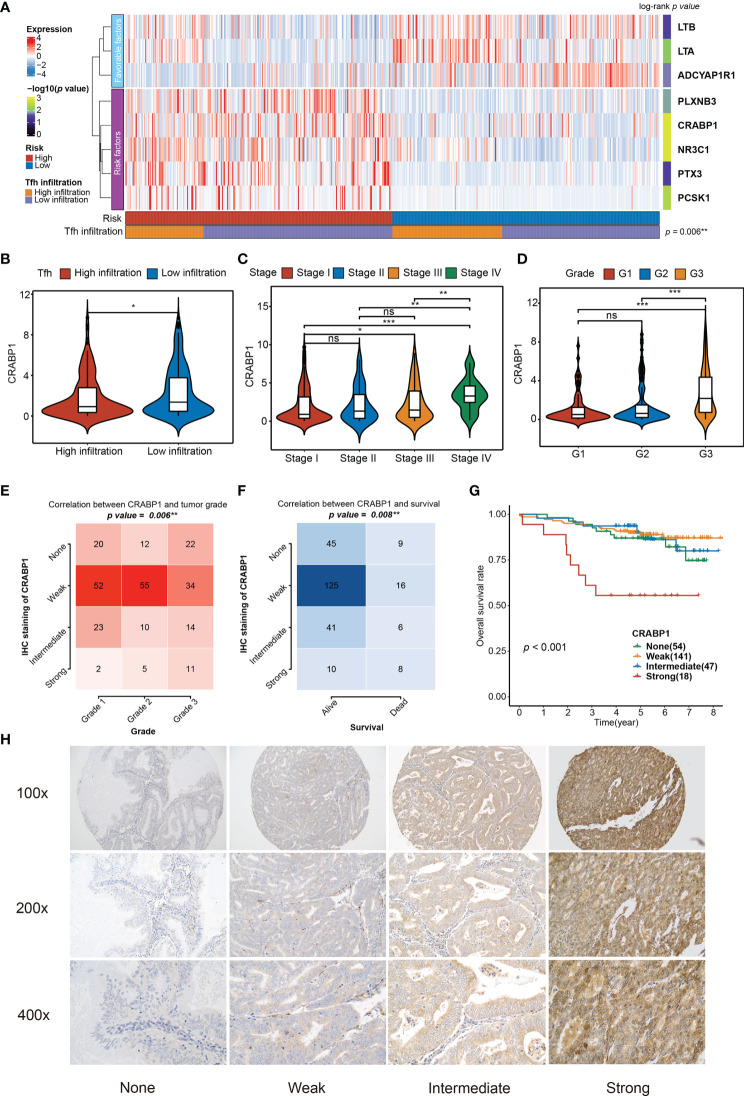
Relationship between the significant genes in the TIRM and the clinical variables. **(A)** The abundances of eight significant immune-related genes (involved in TIRM) in high- and low-risk groups of 520 EC patients. Statistical differences in Tfh infiltration between the two high- and low-risk groups were compared using Fisher’s exact test. The log-rank *p*-values for the univariate Cox regression analysis are shown on the right side of the heatmap, indicated by the color gradient. **p < 0.01. **(B–D)** Distribution of the CRABP1 expression in Tfh infiltration subtypes (high and low) **(B)**, stage (stage I, stage II, stage III, and stage IV) **(C)**, and grade (G1, G2, and G3) **(D)**. Middle line: median; box edges: 25th and 75th percentiles, whiskers: most extreme points. *p < 0.05, **p < 0.01, ***p < 0.001, Wilcoxon rank-sum test and Kruskal–Wallis test. **(E, F)** The correlation between the grade **(E)** and survival status **(F)** and CRABP1 IHC staining in 260 EC tissues (*p* = 0.006 and *p* = 0.008, respectively, Fisher’s exact test). **(G)** Kaplan–Meier curve of the overall survival for 260 EC patients with none, weak, intermediate, and strong CRABP1 IHC staining (*p* < 0.001). **(H)** The CRABP1 immunoreactivity in none, weak, intermediate, and strong scoring at 100×, 200×, and 400× magnifications. ns, non significant.

Among the eight significant genes*, LTB, LTA*, and *ADCYAP1R1* were associated with a favorable prognosis, while the remaining genes, *PLXNB3, CRABP1, NR3C1, PTX3*, and *PCSK1*, were considered to be the risk factors (**Figure 6A**). Particularly, the high Tfh infiltration markers were more enriched in the low-risk group (Fisher’s exact test, *p* = 0.006), which is consistent with the observation that high Tfh was significantly associated with better prognosis (**Figures 2C, D**). Since the TIRM seemed to have a significant prognostic impact in EC, we performed further analysis of the eight involved genes. We identified that *LTA*, *LTB*, and *ADCYAP1R1* expressions were significantly positively associated with Tfh infiltration in favorable genes (Wilcoxon rank-sum test, *p* = 0.006), but not associated with clinical stage of *LTB* (Kruskal–Wallis test, *p* > 0.05) and tumor grade of *LTA* (Kruskal–Wallis test, *p* > 0.05) (**Figure S12**). In contrast, for the five risk factors, we found that the expression level of *PLXNB3, PTX3*, and *CRABP1* were negatively correlated with increased Tfh infiltration (Wilcoxon rank-sum test, *p* < 0.05), but not with NR3C1 and PCSK1 (**Figure 6B** and **Figure S12A**), among which only CRABP1 was significantly associated with both high stage (Stage IV) and grade (G3) (**Figures 6C, D**; **Figures S12B, C**) (Kruskal–Wallis test, *p* < 0.01). Taken together, CRABP1, among the considered parameters, was the most important risk factor among these eight genes. Furthermore, we analyzed the correlations between the *CRABP1* expression and Tfh score. As shown in **Figure S13A**, Tfh was significantly and negatively associated with CRABP1 expression in EC (Spearman rank correlation, *r* = 0.15, *p* = 0.001).

Given that in biological and therapeutic systems, upregulated genes are more likely to be tampered with than downregulated genes, we decided to focus our validation study on CRABP1. To verify the prognostic impact of CRABP1, the potential biomarker, a Karolinska cohort of 260 EC patients with full clinical data was retrieved for validation. We found significant associations between CRABP1 immunoreactivity and poor prognosis (Fisher’s exact test, *p* = 0.006 for grade and *p* = 0.008 for survival status) (**Figures 6E, F**); however, we did not observe any significant difference in tumor stage (Fisher’s exact test, *p* = 0.205, **Figure S13B**). Furthermore, we verified the expression level of CRABP1 in Karolinska cohort by Immunohistochemistry. Patients with strong *CRABP1* staining had the worst OS rate compared to those with none, weak, and intermediate staining (log-rank test, *p* = 0.001, **Figure 6G**). The *CRABP1* immunoreactivity in negative, weak, and moderate scoring at 100×, 200×, and 400× magnifications are shown in **Figure 6H**.

These results strongly suggest that *CRABP1* could be an important prognostic biomarker for EC and the absence of immunoreactivity may indicate favorable treatment effect.

## Discussion

In this study, high immune infiltration was associated with favorable prognosis, which was consistent with previous results, which showed EC tumors with high TIL had improved survival (49, 50). However, the immune infiltration-related gene signature did not predict survival in either training or validation cohort. Although high immune infiltration was significantly associated with high abundance of TIL, the classification of immune infiltration did not correlate with MSI-high and TMB. Our results suggest that immune infiltration classification might be not ideal for the development of prognostic or predictive signatures for immunotherapy in EC. Tumor-associated immune infiltration is a complex event involving heterogeneous immune cell populations. CD8+ cytotoxic T cells, helper T1 cells, and Tfh cells are generally correlated with favorable survival and responsiveness of ICIs, while abundant Treg and M2-like macrophages in tumor microenvironment favor poor prognosis (51). Hence, measuring immune infiltration as a whole activity of TIME might not reflect the different immune cell composition and their distinct impact on the immune contexture.

The current consensus indicates that the Tfh–B cell axis in the tertiary lymphoid structures (TLS) within tumors plays an important role in anti-tumorigenic effects (52). ICI treatment may restore both humoral immune responses and cytotoxic T cell activity against tumor neoantigens mediated by Tfh by elevating interleukin-21 (IL-21) secretion to B cells and CD8+ T cells, respectively (53). High Tfh infiltration in EC tumors correlated with higher IFN-gamma score and CYT activity as well as PD-1/PD-L1 expression, indicating an exhausted T state by chronic antigen exposure (54). Correspondingly, higher TMB and more frequent MSI-H were observed in tumors with high Tfh infiltration, supporting that high mutational load may increase the number of immunogenic neoantigens. Furthermore, high Tfh infiltration was associated with anti-PD-L1 responsiveness in two independent melanoma cohorts (16, 39). These results provide rationale for exploring the utility of Tfh infiltration to select patients eligible for ICI-based therapy.

MSI-H and high TMB levels are biomarkers that support anti-PD-1 immunotherapy in patients with EC (4, 46). However, MSS or pMMR does not necessarily preclude the use of ICI therapies. A phase II trial (KEYNOTE-146) and a confirmatory phase III trial (KEYNOTE-775/Study 309) evaluating the combination treatment of pembrolizumab and lenvatinib reported an ORR of 36% and 30% in patients with pMMR EC progressive on platinum-based chemotherapy, respectively (5). Of note, the ORR of lenvatinib alone in pretreated patients with advanced EC was only 14.3% (55). Dostarlimab (a PD-1 monoclonal antibody) administration alone showed an ORR of 13.4% in patients with pMMR tumors (56). These results raised a critical question: how to identify eligible patients in both p53wt (copy number low) and p53abn (copy number high) subgroups, which are thought to have neither MSI-H nor high TMB. Abundance of TIL may provide a guidance in these patients. In a study using multiplex IHC to estimate TIL in 460 EC tumors, all 5 TIL subsets (CD8+, CD4+, Treg, B cells, and plasma cells) were quantified and clustered into 2 major patterns: TIL-high and TIL-low (11). Although TIL-high tumors were common in POLE mutation and dMMR subtypes, a significant number of p53abn and p53wt tumors also presented with TIL-high pattern. Similarly, in our study, high Tfh infiltration was more prevalent in POLE mutation and dMMR subtypes, but also seen in a minority of tumors with copy number low or copy number high. Additionally, we observed the strong relationship between Tfh score and TIL abundance in EC. There may be merit in prospectively investigating whether TIL, especially Tfh in the TLS within tumor, could be the immune biomarker for identifying candidates for ICI therapy regardless of MSI status.

The presence of TIL has been linked with improved prognosis in various cancer types such as breast cancer and non-small cell lung cancer (NSCLC) (57, 58). The role of TIL in predicting prognosis of EC is somewhat controversial. Previous studies reported TIL as an independent prognostic factor in EC (49, 59). However, TIL did not show independent prognostic significance in multivariable adjustment for variables including the ProMiSE subtype (11). The conflicting findings may be due to the complexity of TIL studies, including differences in measuring and analysis, and stromal or intratumor compartment or both. Moreover, it is necessary to distinguish between the different subpopulations of lymphocytes for their differing roles in modulating cancer progression (51). Through analysis of the TCGA UCEC cohort, we identified Tfh cells as the only immune cell type associated with improved EC survival. Accordingly, Tfh cells have been shown to predict increased survival in NSCLC, colon cancer, and breast cancer (23, 60). To our knowledge, this is the first study to highlight the potential prognostic role of Tfh in EC.

While the histology type, grade, and pathological stage remain paramount for determining the prognosis, molecular classification of EC with TCGA-based classifiers such as ProMisE provided a validated genomic signature to assist clinicians for prognosis estimation (61). According to the Tfh-based classification, we developed and validated an immune-based prognostic signature in EC. We further developed a nomogram combining clinicopathological factors with Tfh risk scores as an individualized tool for assessment of OS in EC. The DCA demonstrated that the combined nomogram was superior to clinicopathological factors or TCGA classification alone. Therefore, incorporating immune-based signature into the traditional clinicopathological features may provide more precise prognostic information.

High CNV was associated with low Tfh infiltration in EC, and *vice versa*. These findings were consistent with previous studies demonstrating that high CNV correlated with high proliferation signature and low immune signature across several cancer types (62, 63). Therefore, high CNV might serve as a negative predictive biomarker for immunotherapy in EC.

CRABP1 functions as a specific binding protein for retinoids to stimulate differentiation but inhibit proliferation. The role of CRABP1 in tumorigenesis is relatively unknown, with reports of both up- and downregulation in different cancer types (64–68). The expression and prognostic significance of CRABP1 has not been previously studied in EC. Our results validated the association between the strong expression of CRABP1 and poor prognosis, which suggests CRABP1 as a prognostic biomarker of EC. Although CRABP1 has been shown to promote cancer progression independent of the retinoid acid binding activity (67), the mechanisms underlying the aggressiveness of CRABP1 in EC remain to be determined.

Although bioinformatics approaches for analyzing bulk RNA sequencing data from the TCGA database have been well established, this study shares some limitations with previous bioinformatics studies. We applied well-validated ssGSEA algorithm to identify immune cell types. However, we did not validate the prognostic value of Tfh using in-house tumor tissues. Given that we used the TMA cores but not whole sections, we were unable to comprehensively assess the extent of Tfh cells mainly in the TLS. Second, the true predictive effect of the Tfh-based gene signature needs to be estimated in a prospective study. Finally, the TCGA UCEC cohort was collected prior to the wide use of immunotherapy. Therefore, these data may not reflect patients receiving immunotherapy. The utility of this immune-based prognostic signature has to be estimated in future cohorts.

In summary, our study identifies the critical role of Tfh in EC. Tfh infiltration-based classification could serve as a predictive biomarker for PD-1 therapy. Through this immune-related classification, we identified and validated an eight-gene prognostic signature. We also developed a risk nomogram, including our Tfh risk scores and clinicopathological factors, and validated that its prognostic utility was superior to that of clinicopathological factors alone or TCGA-based classification. Additionally, we identified and validated the protumorigenic role of CRABP1, which merits further investigation. Overall, our results provide new insights into the TIME of EC along with a strong rationale for individualized assessment and prognosis using Tfh classification.

## Data Availability Statement

The datasets presented in this study can be found in the article and supplemental files. The names of the repositories can be found in the material and method and the relevant links/accession numbers can be found below: https://www.ncbi.nlm.nih.gov/projects/gap/cgi-bin/study.cgi?study_id=phs000452.v2.p1, https://www.ncbi.nlm.nih.gov/geo/query/acc.cgi?acc=GSE91061 and https://ega-archive.org/studies/EGAS00001002556.

## Ethics Statement

The studies involving human participants were reviewed and approved by Karolinska Hospital. Written informed consent for participation was not required for this study in accordance with the national legislation and the institutional requirements.

## Author Contributions

YC and W-KH conceived study design. JL, YZ, JC, and GK collected data. SY, JL, YZ, W-KH, and YC participated in data analysis. YC, SY, JL, and W-KH drafted the paper. EE, JC, and FH participated in revision of the paper. All authors contributed to the article and approved the submitted version.

## Funding

This work was supported by the Swedish Cancer Society, the Swedish Childhood Cancer Foundation, the Cancer Society in Stockholm, and the Stockholm County Council.

## Conflict of Interest

The authors declare that the research was conducted in the absence of any commercial or financial relationships that could be construed as a potential conflict of interest.

## Publisher’s Note

All claims expressed in this article are solely those of the authors and do not necessarily represent those of their affiliated organizations, or those of the publisher, the editors and the reviewers. Any product that may be evaluated in this article, or claim that may be made by its manufacturer, is not guaranteed or endorsed by the publisher.

## Supplementary Material

Supplementary Figure 1
**(A–C)** Kaplan-Meier curve of overall survival rates in EC patients with high- and low-immune **(A)** and stromal **(B)** and tumor purity **(C)** scores (*p = 0.038, 0.39 and 0.428, respectively*). **(C, D)** Distribution of the Immune score in groups with tumor stage (stage I, stage II, stage III, and stage IV) **(C)** and grade (G1, G2, G3) **(D)** Middle line: median; box edges: 25th and 75th percentiles, whiskers: most extreme points. *p < 0.05, Kruskal–Wallis test.

Supplementary Figure 2 Consensus matrices of identified clusters in 520 EC patients with immune signatures (k = 2-10) as well as the cumulative distribution function (CDF) reflect the optimal cluster number.

Supplementary Figure 3
**(A)** Distribution of the tumor purity in groups with high- and low- immune infiltration. Middle line: median; box edges: 25th and 75th percentiles, whiskers: most extreme points. ***p < 0.001, Wilcoxon rank-sum test. **(B)** Kaplan-Meier curve of overall survival rates in EC patients with high- and low-infiltration. (*p* = 0.019). Log-rank test. **(C)** Biological processes and Reactome pathways regulated in the DEGs between high- and low- immune infiltration groups. X axis indicates the most enriched gene sets (-log10(P-value)).

Supplementary Figure 4
**(A)** Partial likelihood deviance under each log (lambda) was drawn in a LASSO Cox regression model. **(B)** The trajectory of each independent gene, the horizontal axis represents the log value of each lambda, and the vertical axis represents the coefficient values. **(C, D)** Kaplan-Meier curve of the overall survival for patients in the immune-based high-risk and low-risk subgroups in the training group **(C)** and validation group **(D)** (p = 0.389 and p = 0.042, respectively).

Supplementary Figure 5 Consensus matrices of identified clusters in 520 EC patients with Tfh signatures (k = 2-10) as well as the cumulative distribution function (CDF) reflect the optimal cluster number.

Supplementary Figure 6 The fraction of age, BMI, race, histological type, stage, grade, survival status, total immune infiltration, and TMB score, showing in high- and low- Tfh infiltration groups.

Supplementary Figure 7
**(A)** Distribution of the immune cell scores in groups with high- and low- Tfh infiltration. Middle line: median; box edges: 25th and 75th percentiles, whiskers: most extreme points. ***p < 0.001, Wilcoxon rank-sum test. **(B)** The correlation between TIL score and Tfh score (Spearman rank correlation).

Supplementary Figure 8
**(A–C)** Distribution of the INS counts (log2 scale) **(A)** DEL counts (log2 scale) **(B)** and SNP counts (log2 scale) **(C)** in groups with high- and low- Tfh infiltration. Middle line: median; box edges: 25th and 75th percentiles, whiskers: most extreme points. ***p < 0.001, Wilcoxon rank-sum test. **(D)** Cumulative CNV regions for high- and low- Tfh infiltration groups. Amplifications are represented in red color, and deletions are represented in blue color. **(E)** The top ten mutated genes between high- and low- Tfh infiltration. **(F)** The Tfh score, TMB score, and CNV frequency of each subtype.

Supplementary Figure 9
**(A, B)** Distribution of the chemokine expressions **(A)** and chemokine receptor expressions **(B)** in groups with high- and low- Tfh infiltration. Middle line: median; box edges: 25th and 75th percentiles, whiskers: most extreme points. * *p* < 0.05; ** *p* < 0.01, *** *p* < 0.001, Wilcoxon rank-sum test.

Supplementary Figure 10
**(A)** The correlations between Tfh score and eight immune inhibitory checkpoint expressions. * p < 0.05; *** p < 0.001. Spearman rank correlation. **(B–D)** The correlations between Tfh score and CTLA4 (B), PD-1 **(C)**, and PD-L1 **(D)** gene expressions. (Spearman rank correlation). **(E, F)** The fraction of the response status of anti-PD-L1 treatment **(E)** and anti-PD-1 treatment **(F)** showing in high- and low- Tfh infiltration groups.

Supplementary Figure 11
**(A)** Volcano plots of the distribution of the DEGs between high- and low- Tfh infiltration groups. The red, and blue color represents upregulated and down-regulated genes, respectively. Genes with |log2FC| > 0.3 are annotated in the plot. **(B)** Partial likelihood deviance under each log (lambda) was drawn in a LASSO Cox regression model. **(C)** The trajectory of each independent gene, the horizontal axis represents the log value of each lambda, and the vertical axis represents the coefficient values. **(D)** ROC curve of the prognostic values of TIRM in all patients in1-, 3- and 5-year OS with AUC = 0.741, 0.759 and 0.786, respectively. **(E)** Kaplan-Meier curve of the overall survival for patients in the Tfh-based high-risk and low-risk subgroups in all patients (*p* < 0.001). **(F–H)** Calibration curves for nomogram in 5-year, 3-year, and 1-year, respectively. **(I)** Net DCA curves shows the net benefits of the nomogram at 1-year OS, the y-axis measures the net benefit. The nomogram has a higher net benefit compared to risk score and simple strategies such as follow-up of all patients (purple line) or no patients (horizontal black line) across the full range of threshold probabilities.

Supplementary Figure 12
**(A–C)** Distribution of the ADCYAP1R1, LTA, LTB, NR3C1, PCSK1, PLXNB3, and PTX3 expression in Tfh infiltration subtypes (high and low) **(A)**, stage (stage I, stage II, stage III, and stage IV) **(B)**, and grade (G1, G2, G3) **(C)**. Middle line: median; box edges: 25th and 75th percentiles, whiskers: most extreme points. *p < 0.05, **p < 0.01, ***p < 0.001, Wilcoxon rank-sum test and Kruskal–Wallis test.

Supplementary Figure 13
**(A)** The correlation between CRABP1 expression and Tfh score (Spearman rank correlation). **(B)** Comparison of the stage and the CRABP1 IHC scores in 260 EC tissues (p = 0.205). Fisher’s exact test.
