# Supplementary material for: Follicular Helper T-Cell-Based Classification of Endometrial Cancer Promotes Precise Checkpoint Immunotherapy and Provides Prognostic Stratification
Source: Front Immunol. 2022 Jan 7;12:788959. doi: 10.3389/fimmu.2021.788959 (PMC8777298; doi:10.3389/fimmu.2021.788959)
Supplement: Supplementary Figure 1 — (A–C) Kaplan-Meier curve of overall survival rates in EC patients with high- and low-immune (A) and stromal (B) and tumor purity (C) scores (p = 0.038, 0.39 and 0.428, respectively). (C, D) Distribution of the Immune score in groups with tumor stage (stage I, stage II, stage III, and stage IV) (C) and grade (G1, G2, G3) (D) Middle line: median; box edges: 25th and 75th percentiles, whiskers: most extreme points. *p < 0.05, Kruskal–Wallis test. [file DataSheet_1.zip › New folder/Figure S13.pdf]

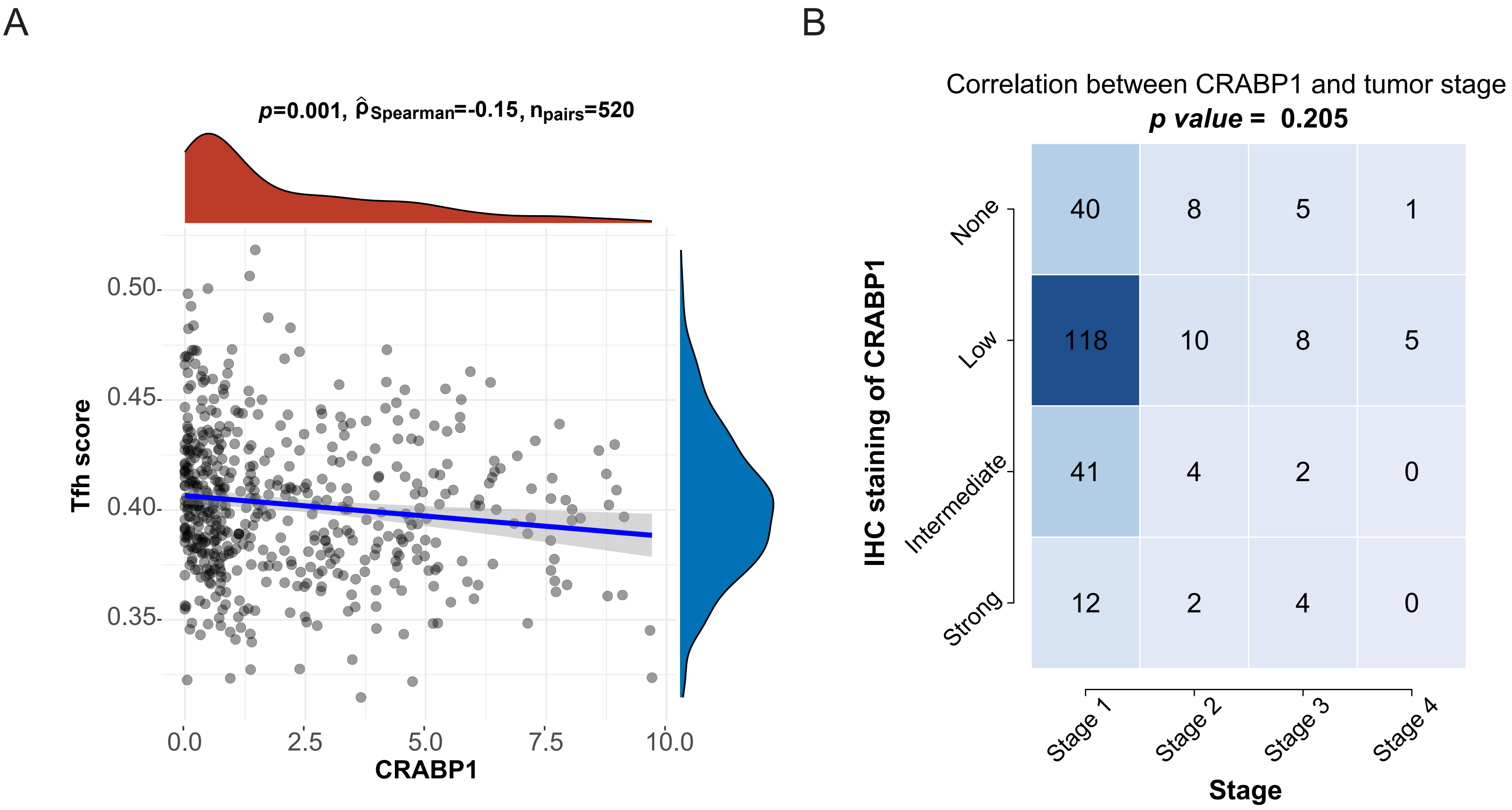
A
p=0.001, ρ̂Spearman=-0.15, npairs=520
Tfh score
0.50
0.45
0.40
0.35
0.0
2.5
5.0
7.5
10.0
CRABP1
B
Correlation between CRABP1 and tumor stage
p value = 0.205
IHC staining of CRABP1
None
Low
Intermediate
Strong
40
8
5
1
118
10
8
5
41
4
2
0
12
2
4
0
Stage 1
Stage 2
Stage 3
Stage 4
Stage
