# Supplementary material for: Follicular Helper T-Cell-Based Classification of Endometrial Cancer Promotes Precise Checkpoint Immunotherapy and Provides Prognostic Stratification
Source: Front Immunol. 2022 Jan 7;12:788959. doi: 10.3389/fimmu.2021.788959 (PMC8777298; doi:10.3389/fimmu.2021.788959)
Supplement: Supplementary Figure 1 — (A–C) Kaplan-Meier curve of overall survival rates in EC patients with high- and low-immune (A) and stromal (B) and tumor purity (C) scores (p = 0.038, 0.39 and 0.428, respectively). (C, D) Distribution of the Immune score in groups with tumor stage (stage I, stage II, stage III, and stage IV) (C) and grade (G1, G2, G3) (D) Middle line: median; box edges: 25th and 75th percentiles, whiskers: most extreme points. *p < 0.05, Kruskal–Wallis test. [file DataSheet_1.zip › New folder/Figure S3.pdf]

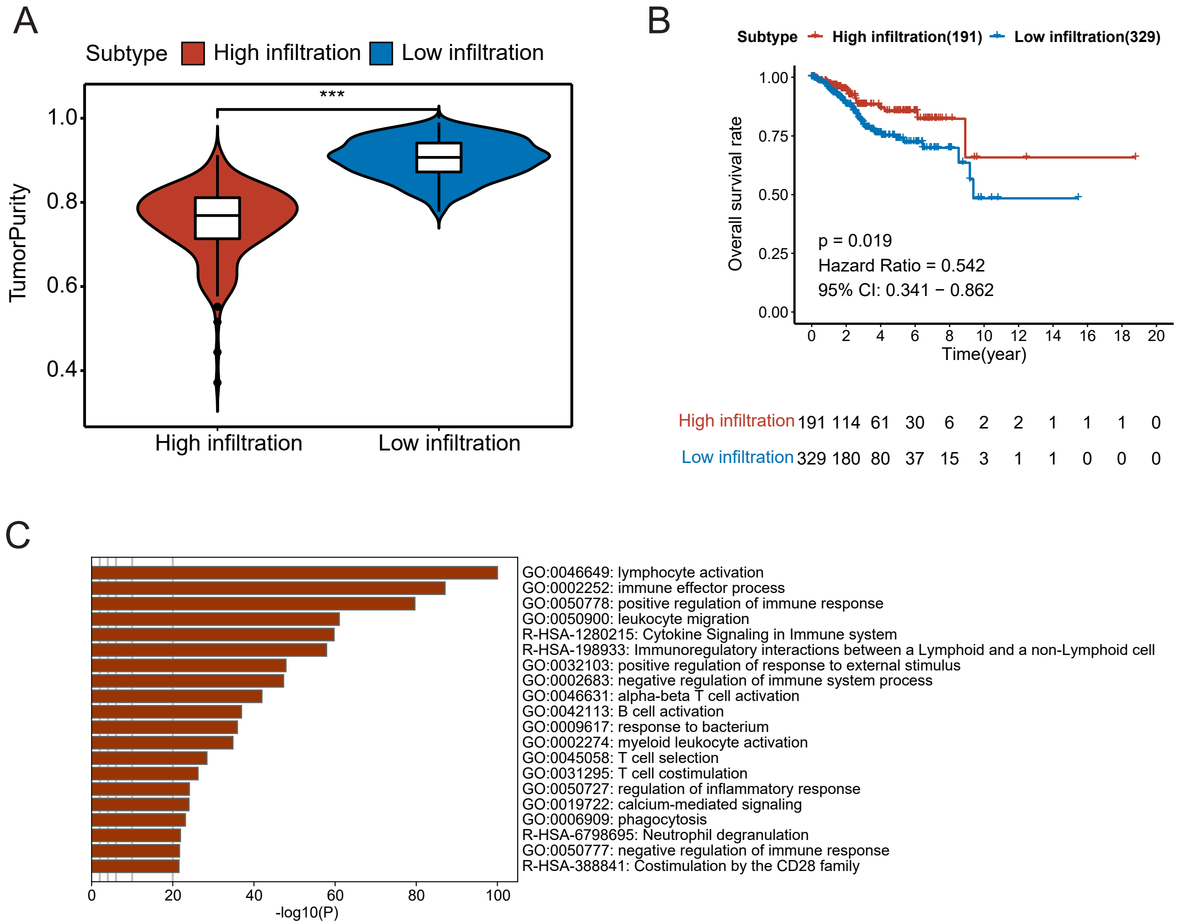
A
Subtype High infiltration Low infiltration
***
TumorPurity
1.0
0.8
0.6
0.4
High infiltration
Low infiltration
B
Subtype High infiltration(191) Low infiltration(329)
Overall survival rate
1.00
0.75
0.50
0.25
0.00
p = 0.019
Hazard Ratio = 0.542
95% CI: 0.341 − 0.862
0 2 4 6 8 10 12 14 16 18 20
Time(year)
High infiltration 191 114 61 30 6 2 2 1 1 1 0
Low infiltration 329 180 80 37 15 3 1 1 0 0 0
C
GO:0046649: lymphocyte activation
GO:0002252: immune effector process
GO:0050778: positive regulation of immune response
GO:0050900: leukocyte migration
R-HSA-1280215: Cytokine Signaling in Immune system
R-HSA-198933: Immunoregulatory interactions between a Lymphoid and a non-Lymphoid cell
GO:0032103: positive regulation of response to external stimulus
GO:0002683: negative regulation of immune system process
GO:0046631: alpha-beta T cell activation
GO:0042113: B cell activation
GO:0009617: response to bacterium
GO:0002274: myeloid leukocyte activation
GO:0045058: T cell selection
GO:0031295: T cell costimulation
GO:0050727: regulation of inflammatory response
GO:0019722: calcium-mediated signaling
GO:0006909: phagocytosis
R-HSA-6798695: Neutrophil degranulation
GO:0050777: negative regulation of immune response
R-HSA-388841: Costimulation by the CD28 family
0 20 40 60 80 100
-log10(P)
