# Supplementary material for: Follicular Helper T-Cell-Based Classification of Endometrial Cancer Promotes Precise Checkpoint Immunotherapy and Provides Prognostic Stratification
Source: Front Immunol. 2022 Jan 7;12:788959. doi: 10.3389/fimmu.2021.788959 (PMC8777298; doi:10.3389/fimmu.2021.788959)
Supplement: Supplementary Figure 1 — (A–C) Kaplan-Meier curve of overall survival rates in EC patients with high- and low-immune (A) and stromal (B) and tumor purity (C) scores (p = 0.038, 0.39 and 0.428, respectively). (C, D) Distribution of the Immune score in groups with tumor stage (stage I, stage II, stage III, and stage IV) (C) and grade (G1, G2, G3) (D) Middle line: median; box edges: 25th and 75th percentiles, whiskers: most extreme points. *p < 0.05, Kruskal–Wallis test. [file DataSheet_1.zip › New folder/Figure S8.pdf]

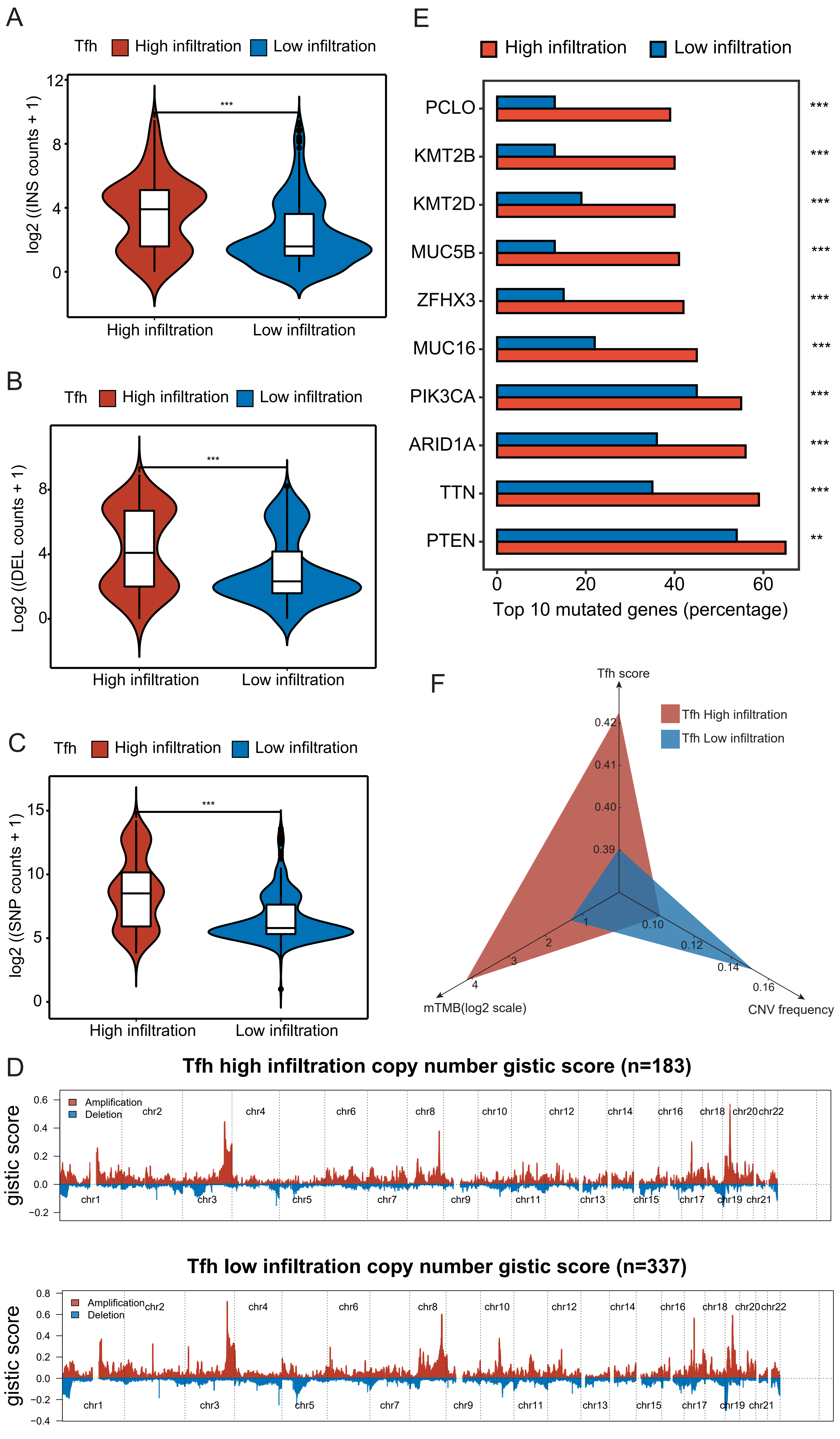

A
Tfh High infiltration Low infiltration
log2 ((INS counts + 1)
***
High infiltration
Low infiltration
B
Tfh High infiltration Low infiltration
Log2 ((DEL counts + 1)
***
High infiltration
Low infiltration
C
Tfh High infiltration Low infiltration
log2 ((SNP counts + 1)
***
High infiltration
Low infiltration
D
Tfh high infiltration copy number gistic score (n=183)
gistic score
Amplification
Deletion
Tfh low infiltration copy number gistic score (n=337)
E
High infiltration Low infiltration
PCLO
KMT2B
KMT2D
MUC5B
ZFHX3
MUC16
PIK3CA
ARID1A
TTN
PTEN
Top 10 mutated genes (percentage)
F
Tfh score
Tfh High infiltration
Tfh Low infiltration
mTMB(log2 scale)
CNV frequency
