# Supplementary material for: Follicular Helper T-Cell-Based Classification of Endometrial Cancer Promotes Precise Checkpoint Immunotherapy and Provides Prognostic Stratification
Source: Front Immunol. 2022 Jan 7;12:788959. doi: 10.3389/fimmu.2021.788959 (PMC8777298; doi:10.3389/fimmu.2021.788959)
Supplement: Supplementary Figure 1 — (A–C) Kaplan-Meier curve of overall survival rates in EC patients with high- and low-immune (A) and stromal (B) and tumor purity (C) scores (p = 0.038, 0.39 and 0.428, respectively). (C, D) Distribution of the Immune score in groups with tumor stage (stage I, stage II, stage III, and stage IV) (C) and grade (G1, G2, G3) (D) Middle line: median; box edges: 25th and 75th percentiles, whiskers: most extreme points. *p < 0.05, Kruskal–Wallis test. [file DataSheet_1.zip › New folder/Table S5.docx]

**Table. S5. The Clinical Features of TCGA-UCEC training group (n= 312) and validation group (n= 208).**

| Clinical  Features | TCGA-UCEC (n=520) | | P-value |
| --- | --- | --- | --- |
|  | Training group (n=312) | Validation group (n=208) |  |
| **Age** |  |  |  |
| <64 | 154 | 99 | 0.213 |
| ≥64  N.A. | 158  0 | 107  2 |  |
| **BMI** |  |  |  |
| <32 | 143 | 96 | 0.982 |
| ≥32  N.A. | 152  17 | 100  12 |  |
| **RACE** |  |  |  |
| American Indian or Alaska native | 2 | 1 | 0.619 |
| Asian  Black or African American  Native Hawaiian or other  Pacific Islander  White  N.A. | 14  56  6  217  17 | 6  47  2  138  14 |  |
| **Histological type** |  |  |  |
| Endometrioid endometrial adenocarcinoma | 227 | 162 | 0.215 |
| Mixed serous and endometrioid  Serous endometrial adenocarcinoma | 16  69 | 5  41 |  |
| **Histological grade** |  |  |  |
| Grade 1 | 65 | 31 | 0.361 |
| Grade 2  Grade 3  N.A. | 68  173  6 | 48  126  3 |  |
| **Clinical stage** |  |  |  |
| Stage I | 196 | 129 | 0.967 |
| Stage II  Stage III  Stage IV | 29  72  15 | 20  47  12 |  |
| **Survival status** |  |  |  |
| Alive | 265 | 179 | 0.723 |
| Dead | 47 | 29 |  |
