# Supplementary figures and images for: Follicular Helper T-Cell-Based Classification of Endometrial Cancer Promotes Precise Checkpoint Immunotherapy and Provides Prognostic Stratification
Source: Front Immunol. 2022 Jan 7;12:788959. doi: 10.3389/fimmu.2021.788959 (PMC8777298; doi:10.3389/fimmu.2021.788959)

A

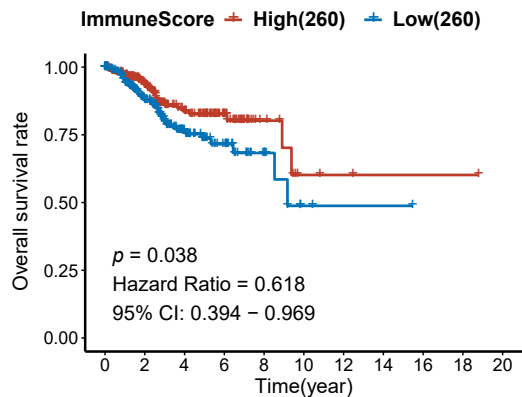

B

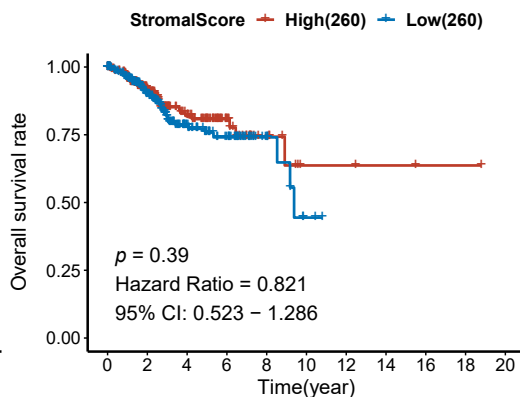

C

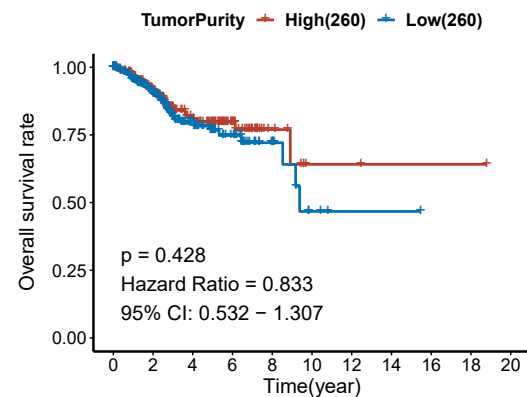

High 260 149 81 38 10 3 2 1 1 1 0  
Low 260 145 60 29 11 2 1 1 0 0 0

High 260 148 74 29 10 3 3 2 1 1 0  
Low 260 146 67 38 11 2 0 0 0 0 0

High 260 146 74 33 8 2 2 1 1 1 0  
Low 260 148 67 34 13 3 1 1 0 0 0

D

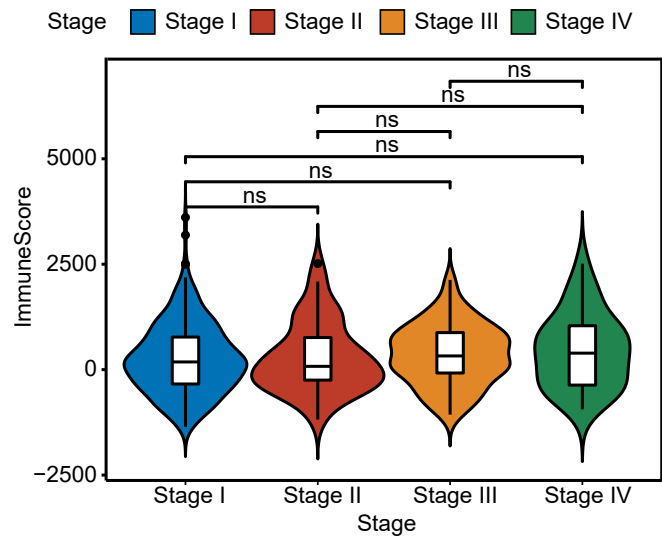

E

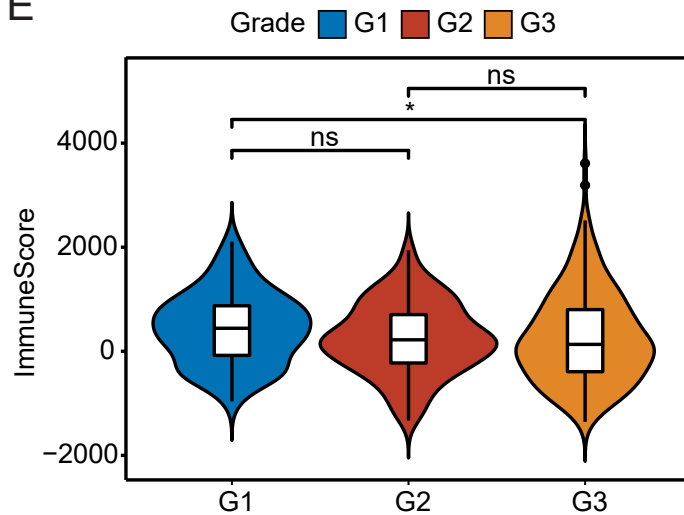

Supplement: Supplementary Figure 1 — (A–C) Kaplan-Meier curve of overall survival rates in EC patients with high- and low-immune (A) and stromal (B) and tumor purity (C) scores (p = 0.038, 0.39 and 0.428, respectively). (C, D) Distribution of the Immune score in groups with tumor stage (stage I, stage II, stage III, and stage IV) (C) and grade (G1, G2, G3) (D) Middle line: median; box edges: 25th and 75th percentiles, whiskers: most extreme points. *p < 0.05, Kruskal–Wallis test. [file DataSheet_1.zip › New folder/Figure S1.pdf]

A

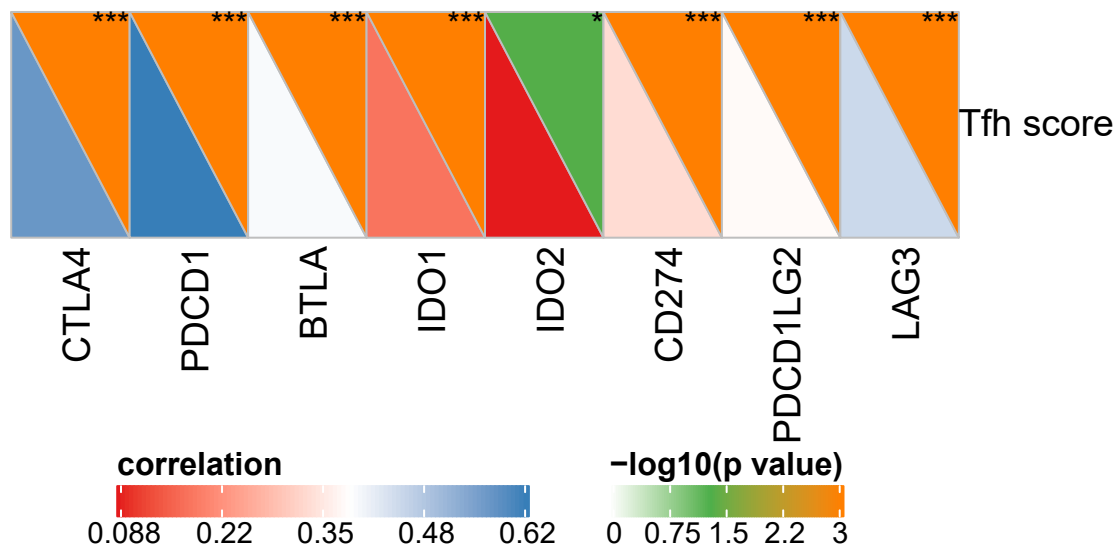

B

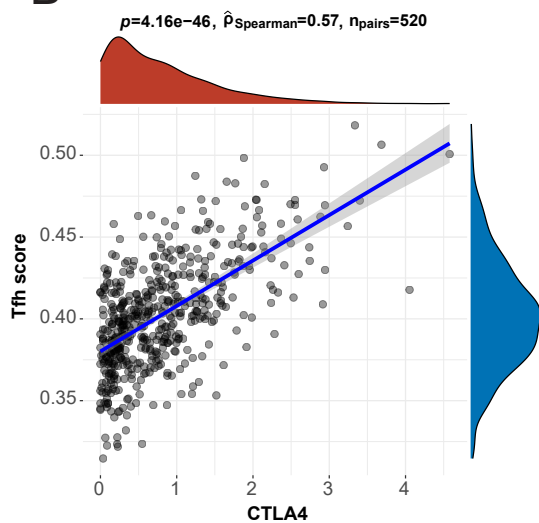

C

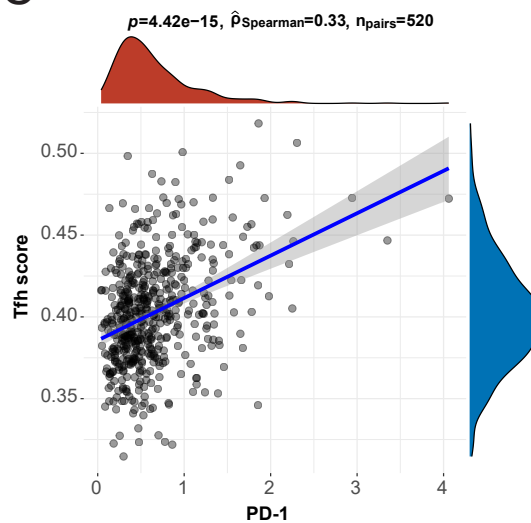

D

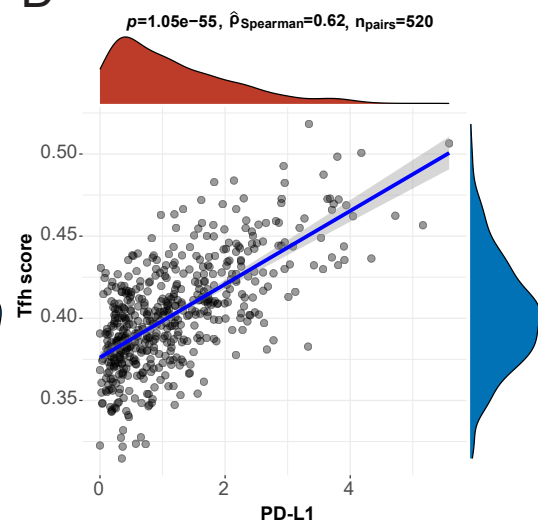

E

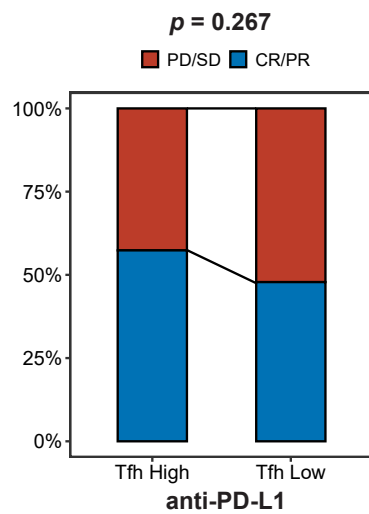

F

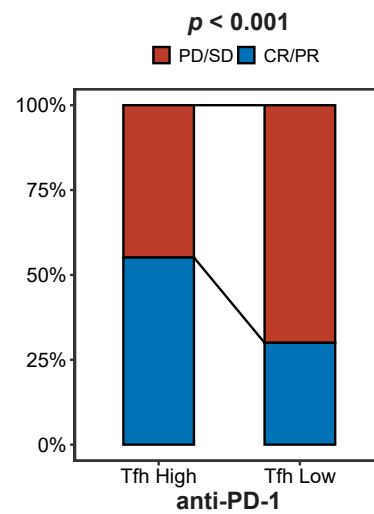

Supplement: Supplementary Figure 1 — (A–C) Kaplan-Meier curve of overall survival rates in EC patients with high- and low-immune (A) and stromal (B) and tumor purity (C) scores (p = 0.038, 0.39 and 0.428, respectively). (C, D) Distribution of the Immune score in groups with tumor stage (stage I, stage II, stage III, and stage IV) (C) and grade (G1, G2, G3) (D) Middle line: median; box edges: 25th and 75th percentiles, whiskers: most extreme points. *p < 0.05, Kruskal–Wallis test. [file DataSheet_1.zip › New folder/Figure S10.pdf]

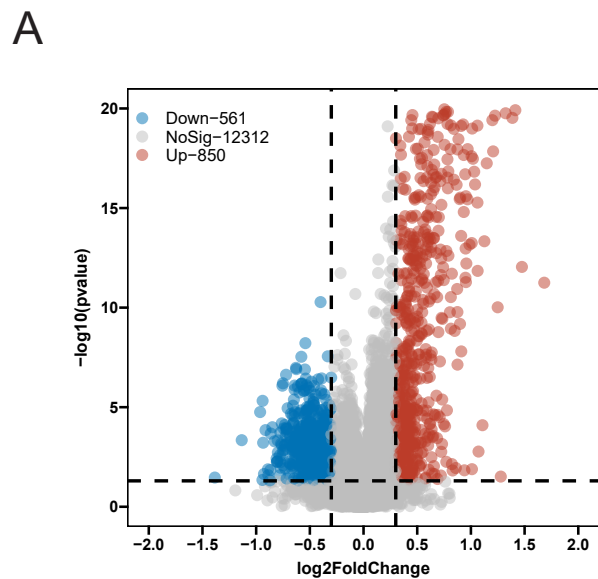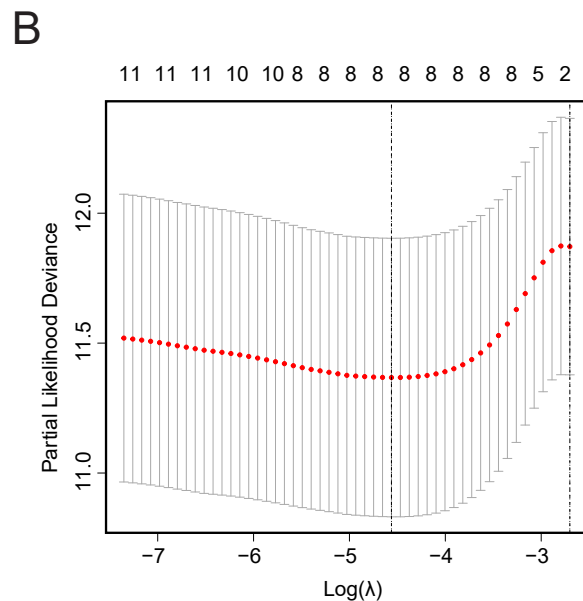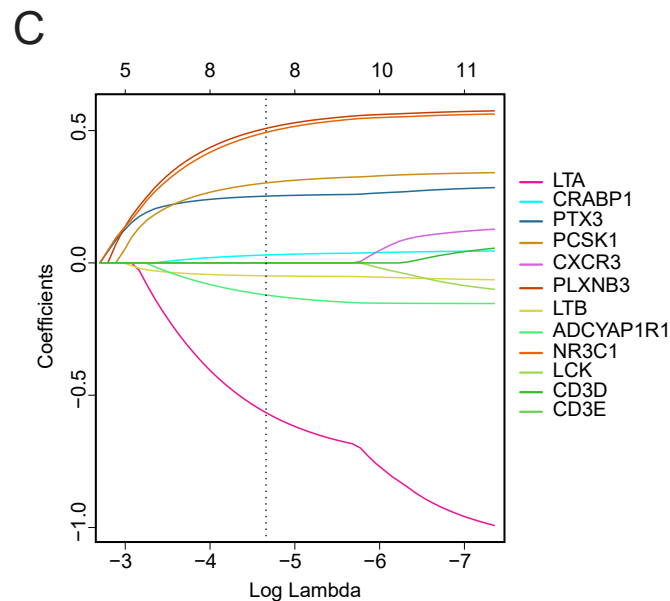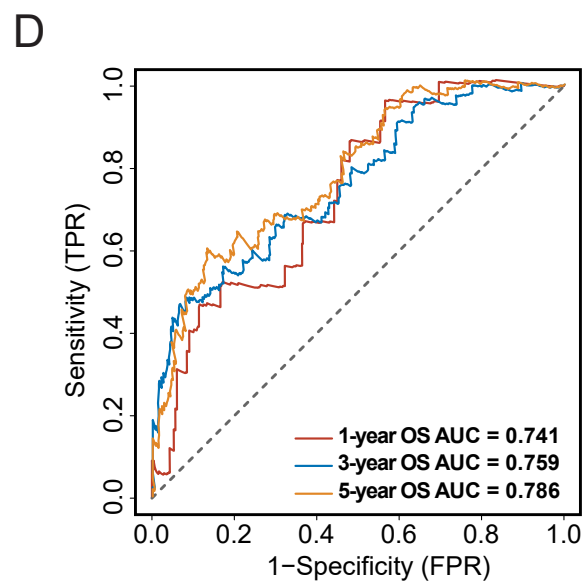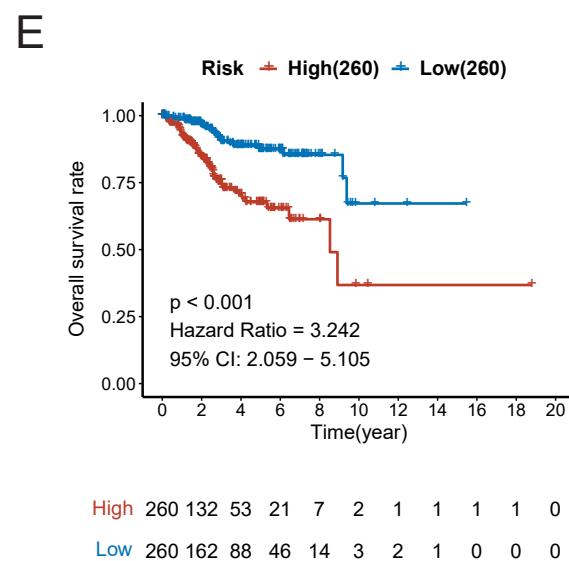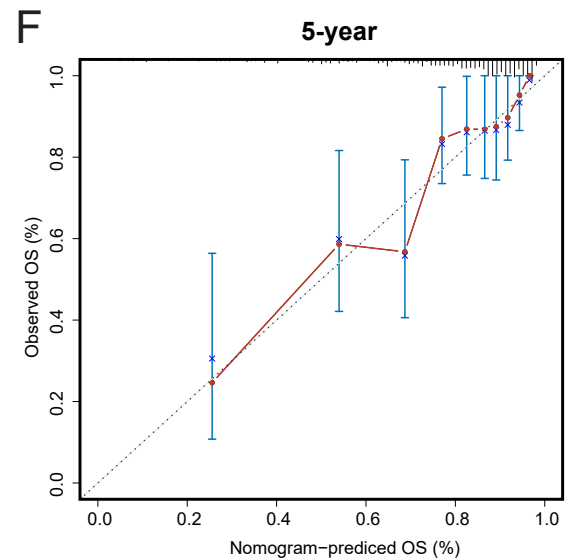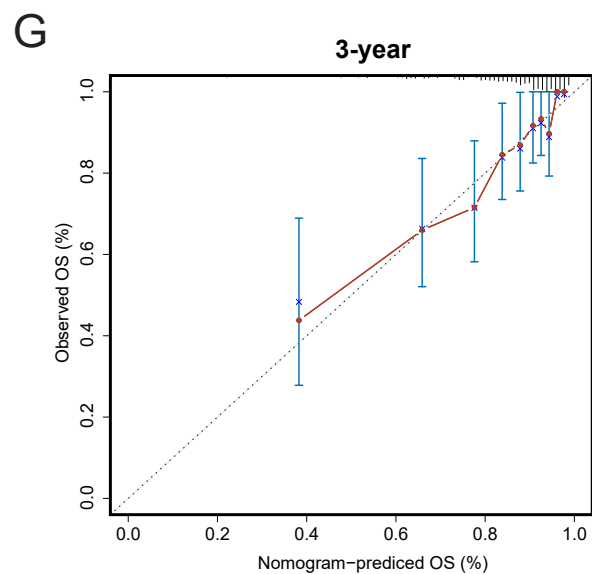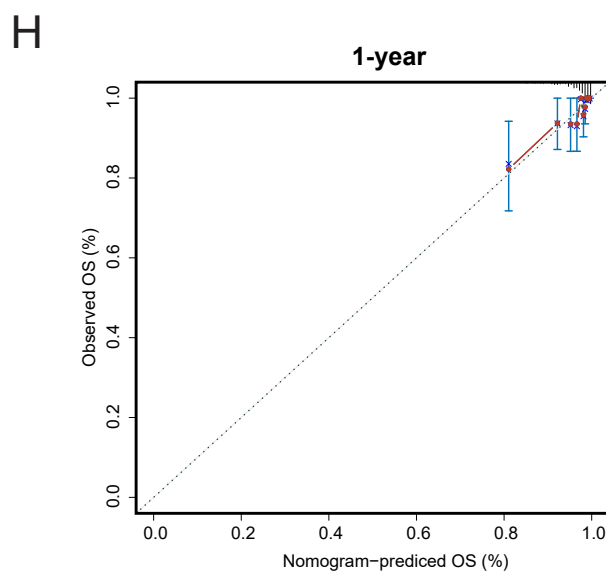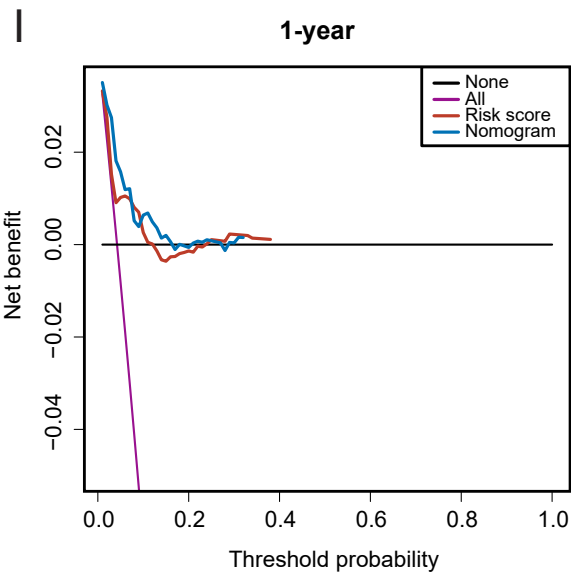

Supplement: Supplementary Figure 1 — (A–C) Kaplan-Meier curve of overall survival rates in EC patients with high- and low-immune (A) and stromal (B) and tumor purity (C) scores (p = 0.038, 0.39 and 0.428, respectively). (C, D) Distribution of the Immune score in groups with tumor stage (stage I, stage II, stage III, and stage IV) (C) and grade (G1, G2, G3) (D) Middle line: median; box edges: 25th and 75th percentiles, whiskers: most extreme points. *p < 0.05, Kruskal–Wallis test. [file DataSheet_1.zip › New folder/Figure S11.pdf]

**A**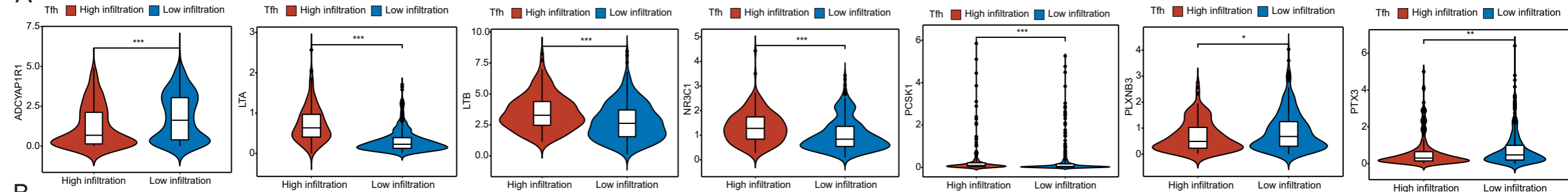**B**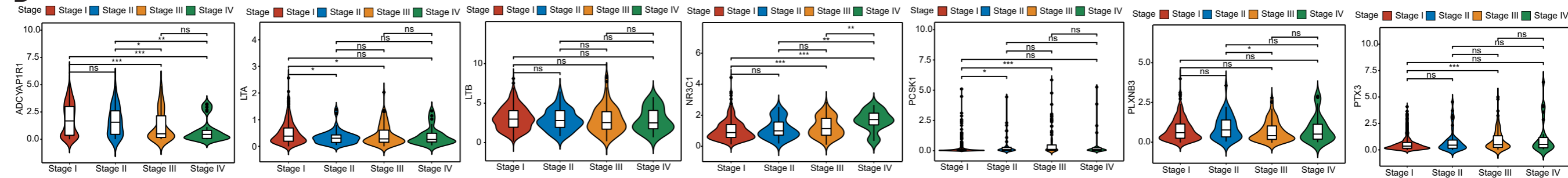**C**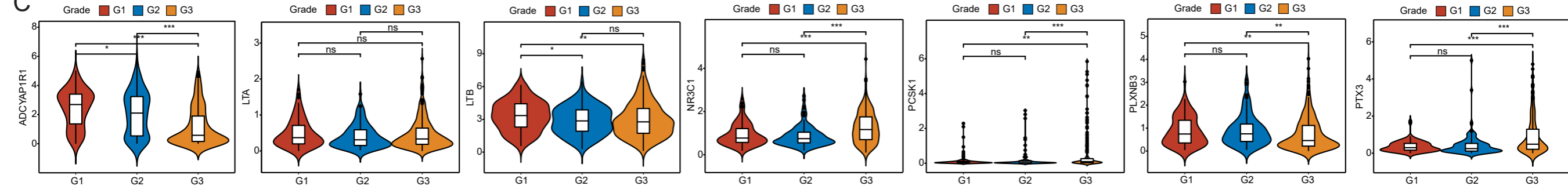

Supplement: Supplementary Figure 1 — (A–C) Kaplan-Meier curve of overall survival rates in EC patients with high- and low-immune (A) and stromal (B) and tumor purity (C) scores (p = 0.038, 0.39 and 0.428, respectively). (C, D) Distribution of the Immune score in groups with tumor stage (stage I, stage II, stage III, and stage IV) (C) and grade (G1, G2, G3) (D) Middle line: median; box edges: 25th and 75th percentiles, whiskers: most extreme points. *p < 0.05, Kruskal–Wallis test. [file DataSheet_1.zip › New folder/Figure S12.pdf]

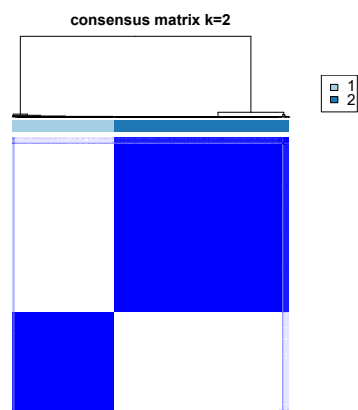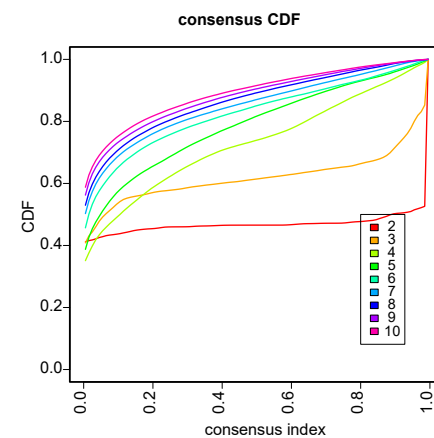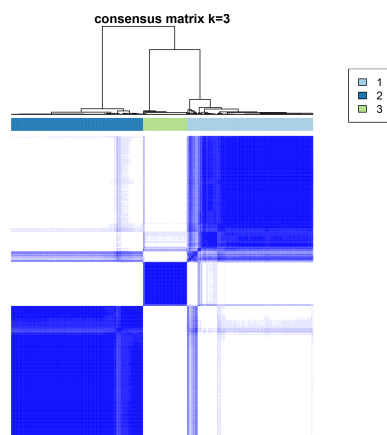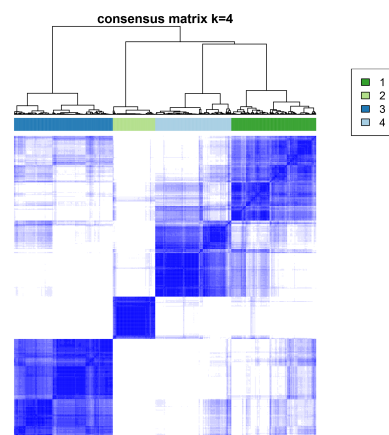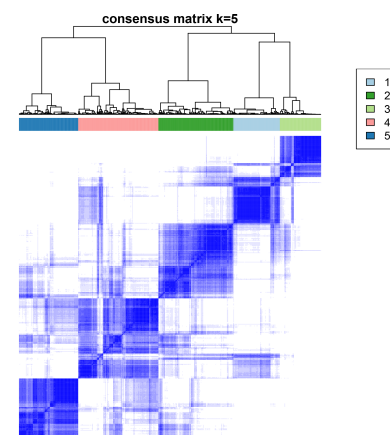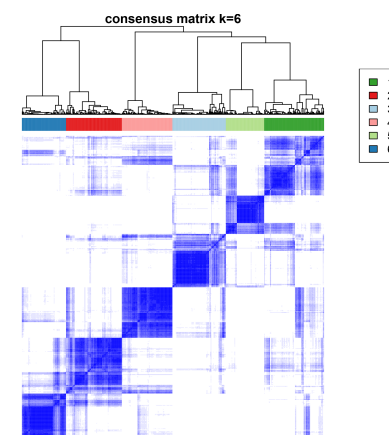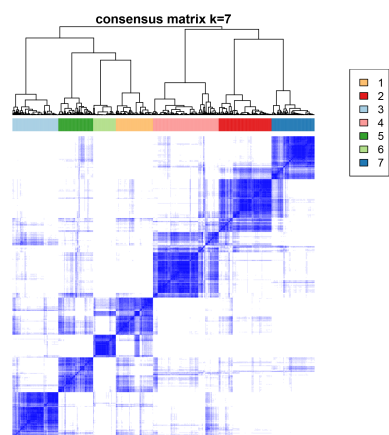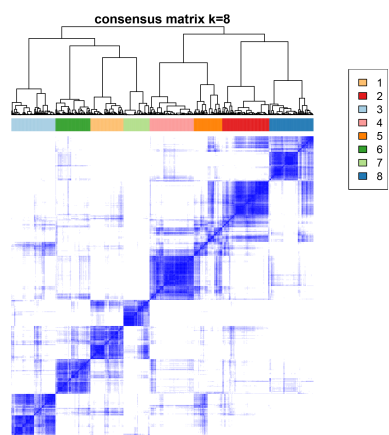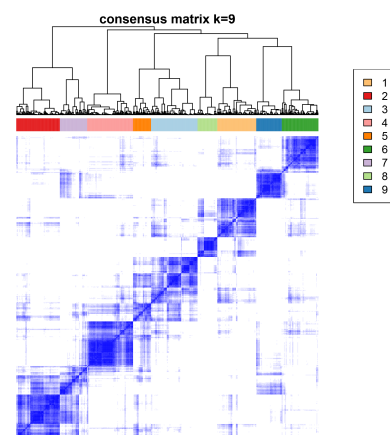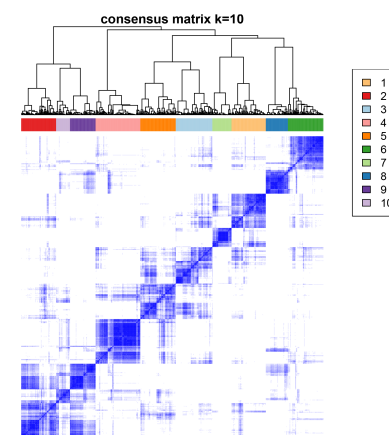

Supplement: Supplementary Figure 1 — (A–C) Kaplan-Meier curve of overall survival rates in EC patients with high- and low-immune (A) and stromal (B) and tumor purity (C) scores (p = 0.038, 0.39 and 0.428, respectively). (C, D) Distribution of the Immune score in groups with tumor stage (stage I, stage II, stage III, and stage IV) (C) and grade (G1, G2, G3) (D) Middle line: median; box edges: 25th and 75th percentiles, whiskers: most extreme points. *p < 0.05, Kruskal–Wallis test. [file DataSheet_1.zip › New folder/Figure S2.pdf]

A

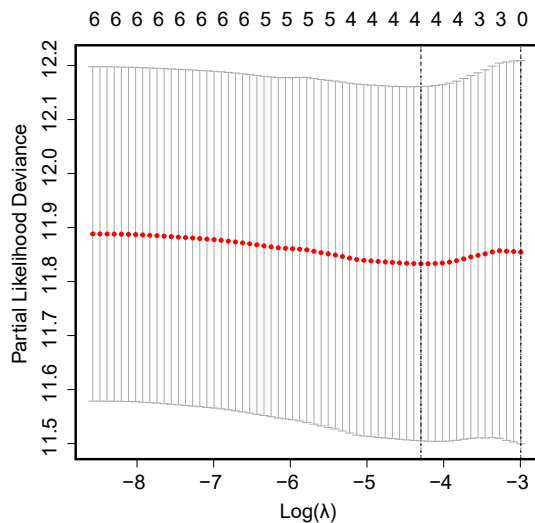

B

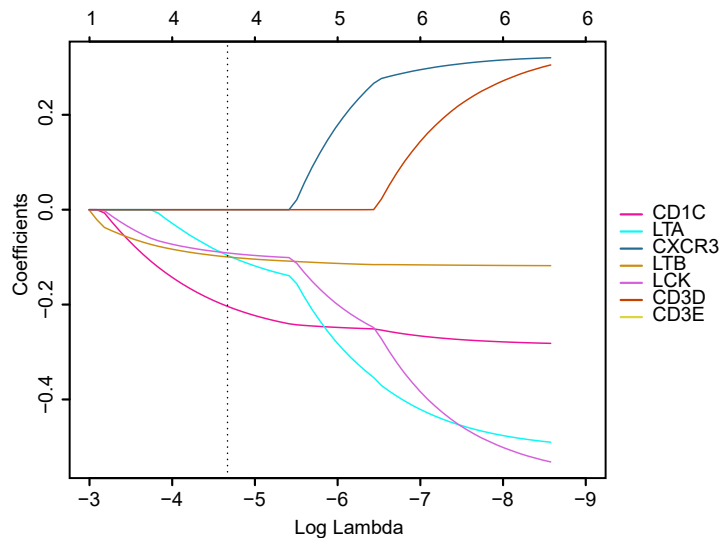

C

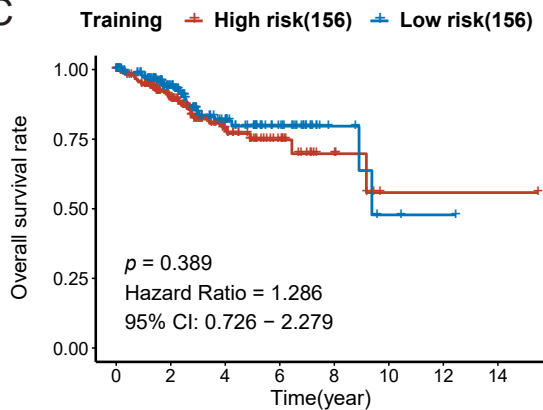

|      |     |    |    |    |   |   |   |   |
|------|-----|----|----|----|---|---|---|---|
| High | 156 | 92 | 42 | 20 | 7 | 1 | 1 | 1 |
| Low  | 156 | 97 | 49 | 25 | 6 | 2 | 1 | 0 |

D

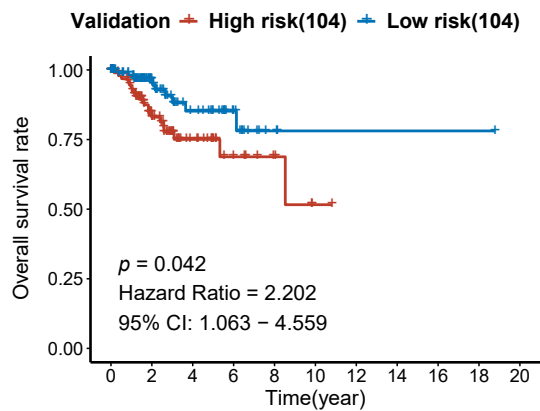

|      |     |    |    |    |   |   |   |   |   |   |
|------|-----|----|----|----|---|---|---|---|---|---|
| High | 104 | 53 | 22 | 9  | 5 | 1 | 0 | 0 | 0 | 0 |
| Low  | 104 | 52 | 28 | 13 | 3 | 1 | 1 | 1 | 1 | 0 |

Supplement: Supplementary Figure 1 — (A–C) Kaplan-Meier curve of overall survival rates in EC patients with high- and low-immune (A) and stromal (B) and tumor purity (C) scores (p = 0.038, 0.39 and 0.428, respectively). (C, D) Distribution of the Immune score in groups with tumor stage (stage I, stage II, stage III, and stage IV) (C) and grade (G1, G2, G3) (D) Middle line: median; box edges: 25th and 75th percentiles, whiskers: most extreme points. *p < 0.05, Kruskal–Wallis test. [file DataSheet_1.zip › New folder/Figure S4.pdf]

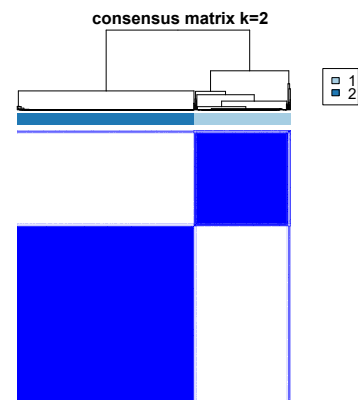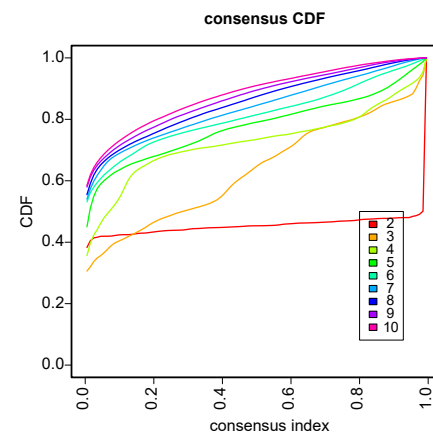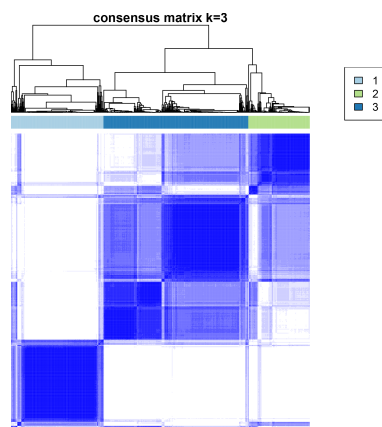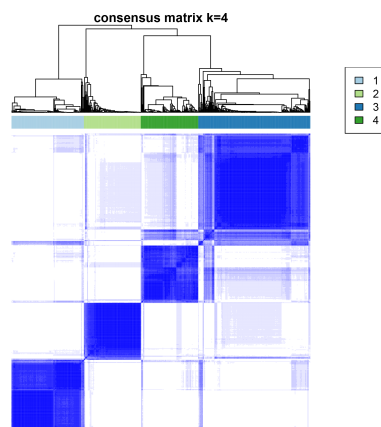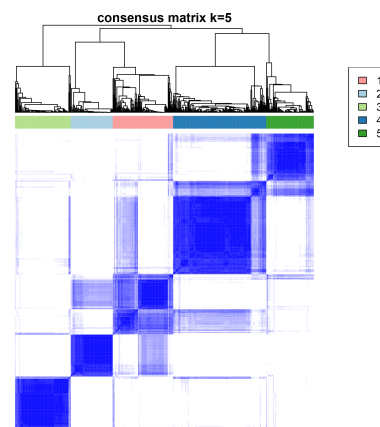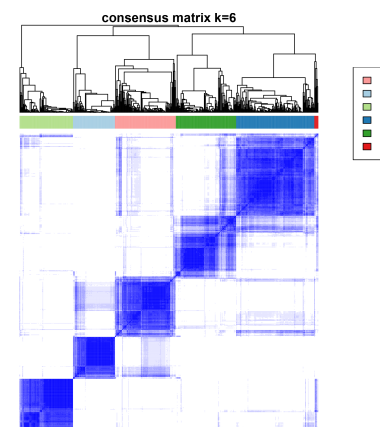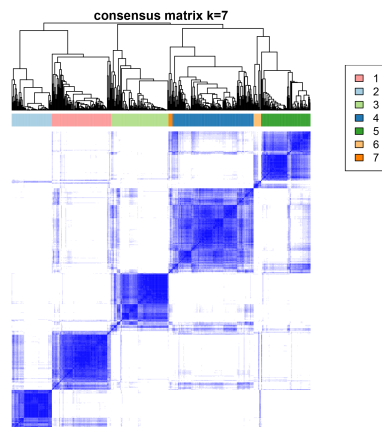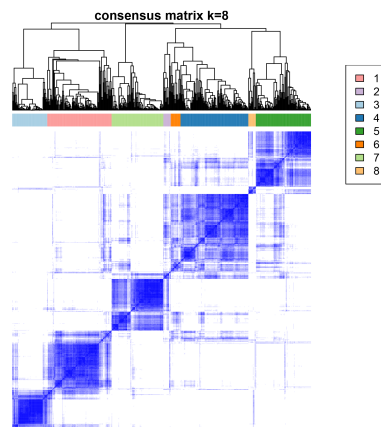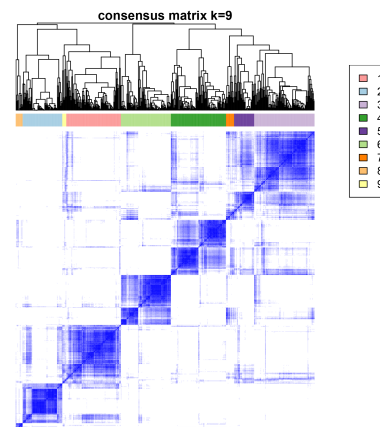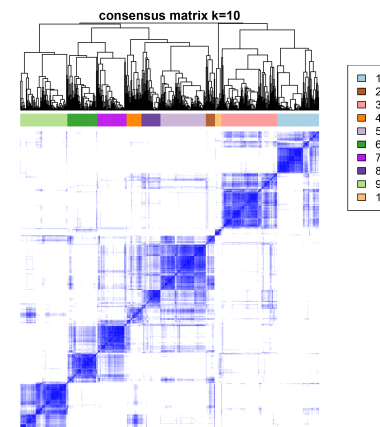

Supplement: Supplementary Figure 1 — (A–C) Kaplan-Meier curve of overall survival rates in EC patients with high- and low-immune (A) and stromal (B) and tumor purity (C) scores (p = 0.038, 0.39 and 0.428, respectively). (C, D) Distribution of the Immune score in groups with tumor stage (stage I, stage II, stage III, and stage IV) (C) and grade (G1, G2, G3) (D) Middle line: median; box edges: 25th and 75th percentiles, whiskers: most extreme points. *p < 0.05, Kruskal–Wallis test. [file DataSheet_1.zip › New folder/Figure S5.pdf]

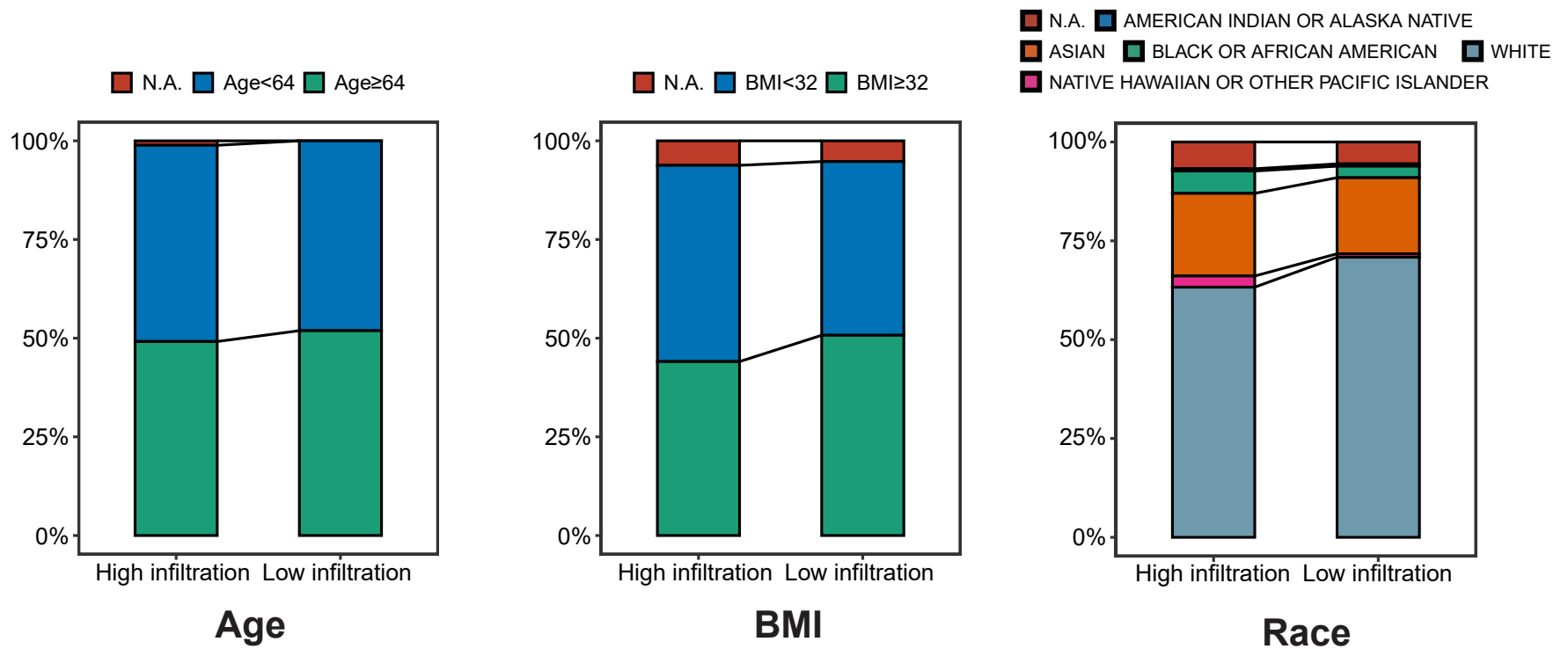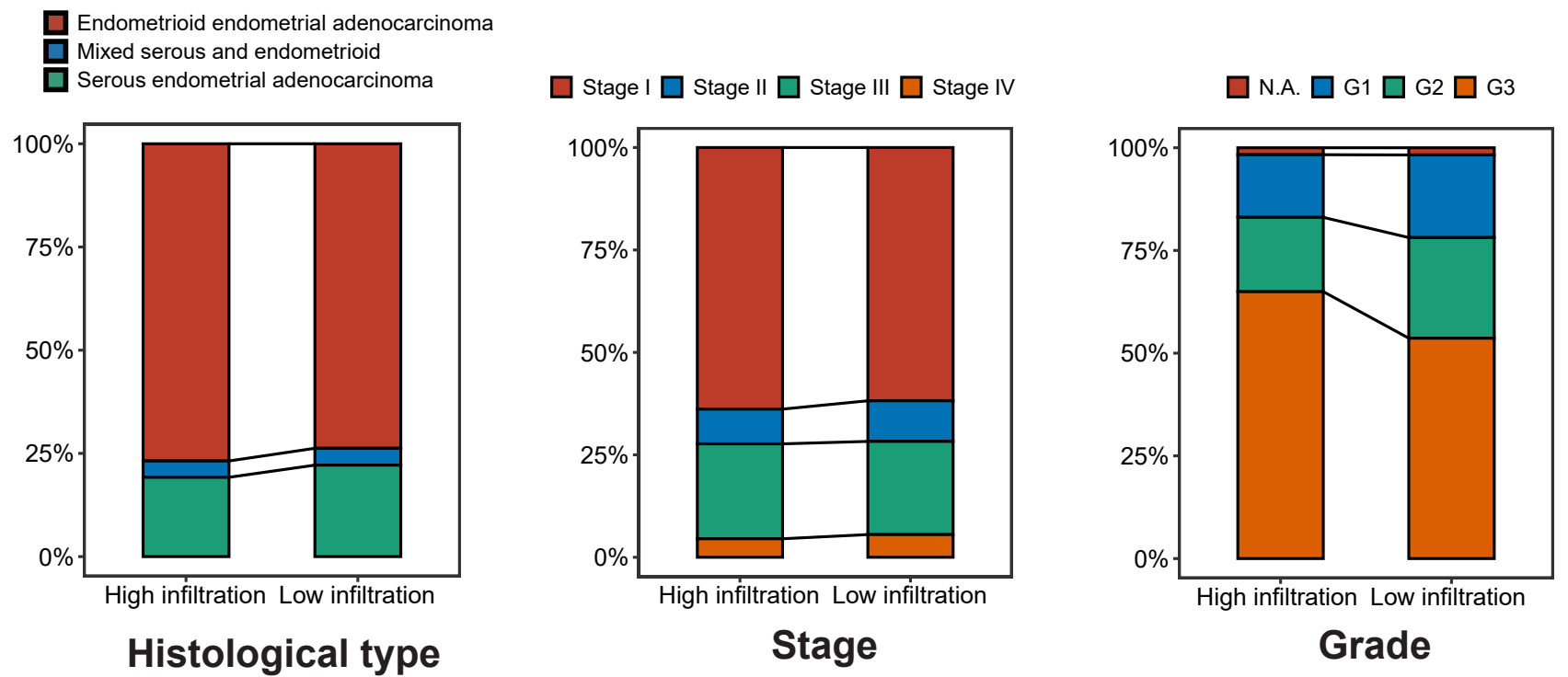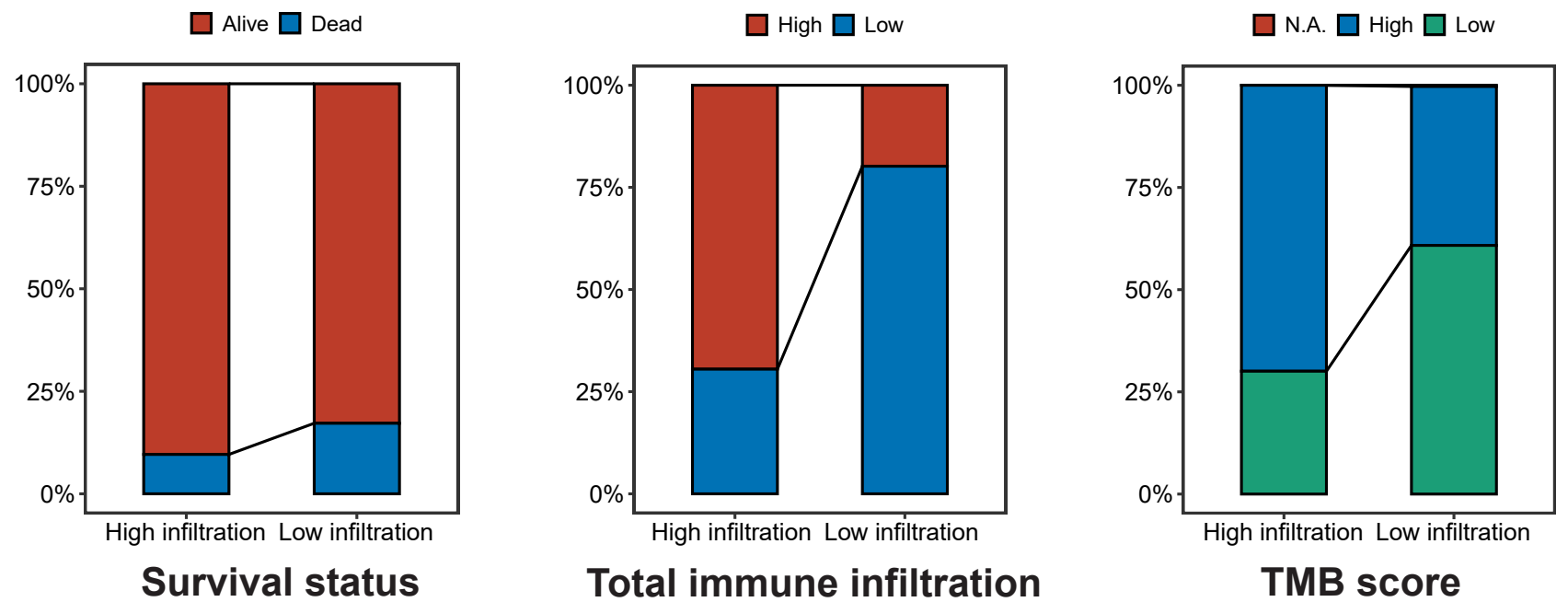

Supplement: Supplementary Figure 1 — (A–C) Kaplan-Meier curve of overall survival rates in EC patients with high- and low-immune (A) and stromal (B) and tumor purity (C) scores (p = 0.038, 0.39 and 0.428, respectively). (C, D) Distribution of the Immune score in groups with tumor stage (stage I, stage II, stage III, and stage IV) (C) and grade (G1, G2, G3) (D) Middle line: median; box edges: 25th and 75th percentiles, whiskers: most extreme points. *p < 0.05, Kruskal–Wallis test. [file DataSheet_1.zip › New folder/Figure S6.pdf]

A

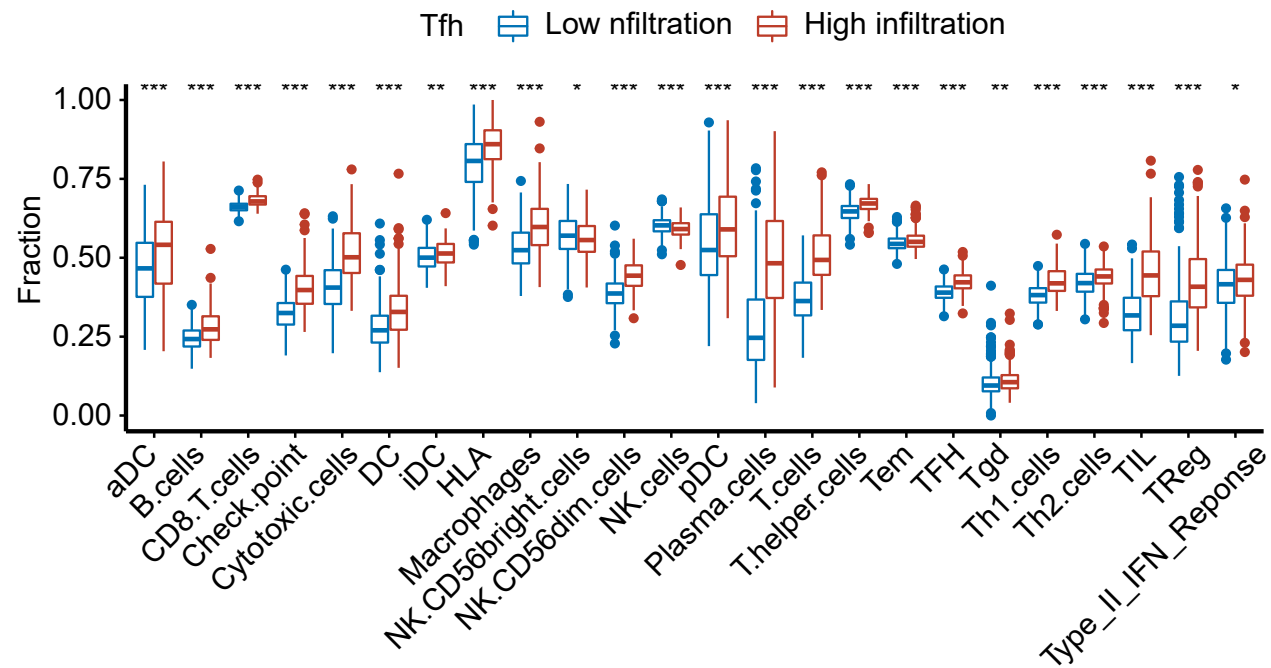

B

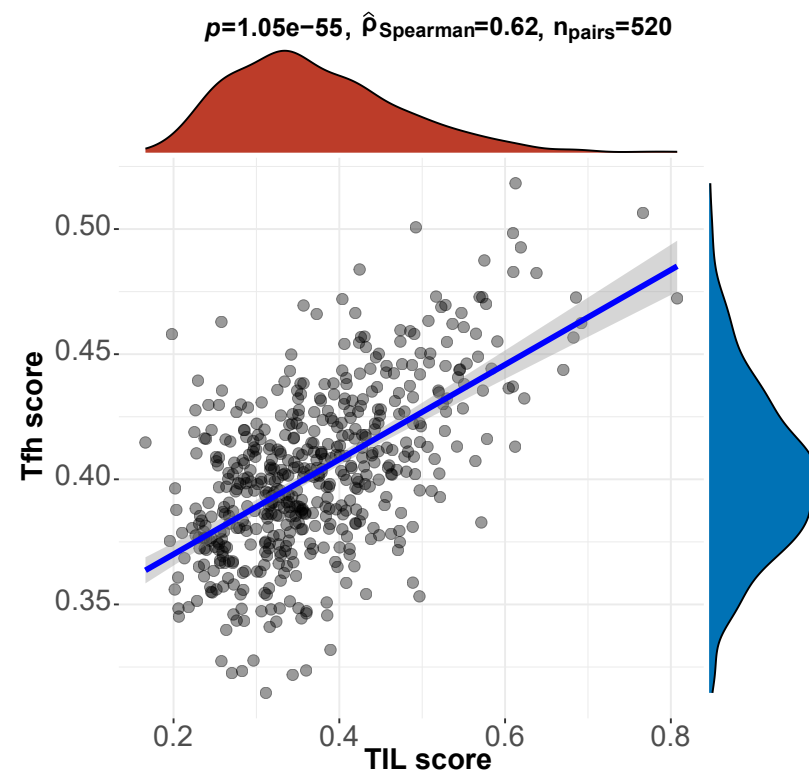

Supplement: Supplementary Figure 1 — (A–C) Kaplan-Meier curve of overall survival rates in EC patients with high- and low-immune (A) and stromal (B) and tumor purity (C) scores (p = 0.038, 0.39 and 0.428, respectively). (C, D) Distribution of the Immune score in groups with tumor stage (stage I, stage II, stage III, and stage IV) (C) and grade (G1, G2, G3) (D) Middle line: median; box edges: 25th and 75th percentiles, whiskers: most extreme points. *p < 0.05, Kruskal–Wallis test. [file DataSheet_1.zip › New folder/Figure S7.pdf]
