# Supplementary material for: Follicular Helper T-Cell-Based Classification of Endometrial Cancer Promotes Precise Checkpoint Immunotherapy and Provides Prognostic Stratification
Source: Front Immunol. 2022 Jan 7;12:788959. doi: 10.3389/fimmu.2021.788959 (PMC8777298; doi:10.3389/fimmu.2021.788959)
Supplement: Supplementary Figure 1 — (A–C) Kaplan-Meier curve of overall survival rates in EC patients with high- and low-immune (A) and stromal (B) and tumor purity (C) scores (p = 0.038, 0.39 and 0.428, respectively). (C, D) Distribution of the Immune score in groups with tumor stage (stage I, stage II, stage III, and stage IV) (C) and grade (G1, G2, G3) (D) Middle line: median; box edges: 25th and 75th percentiles, whiskers: most extreme points. *p < 0.05, Kruskal–Wallis test. [file Figure S9.pdf › New folder/Figure S9.pdf]

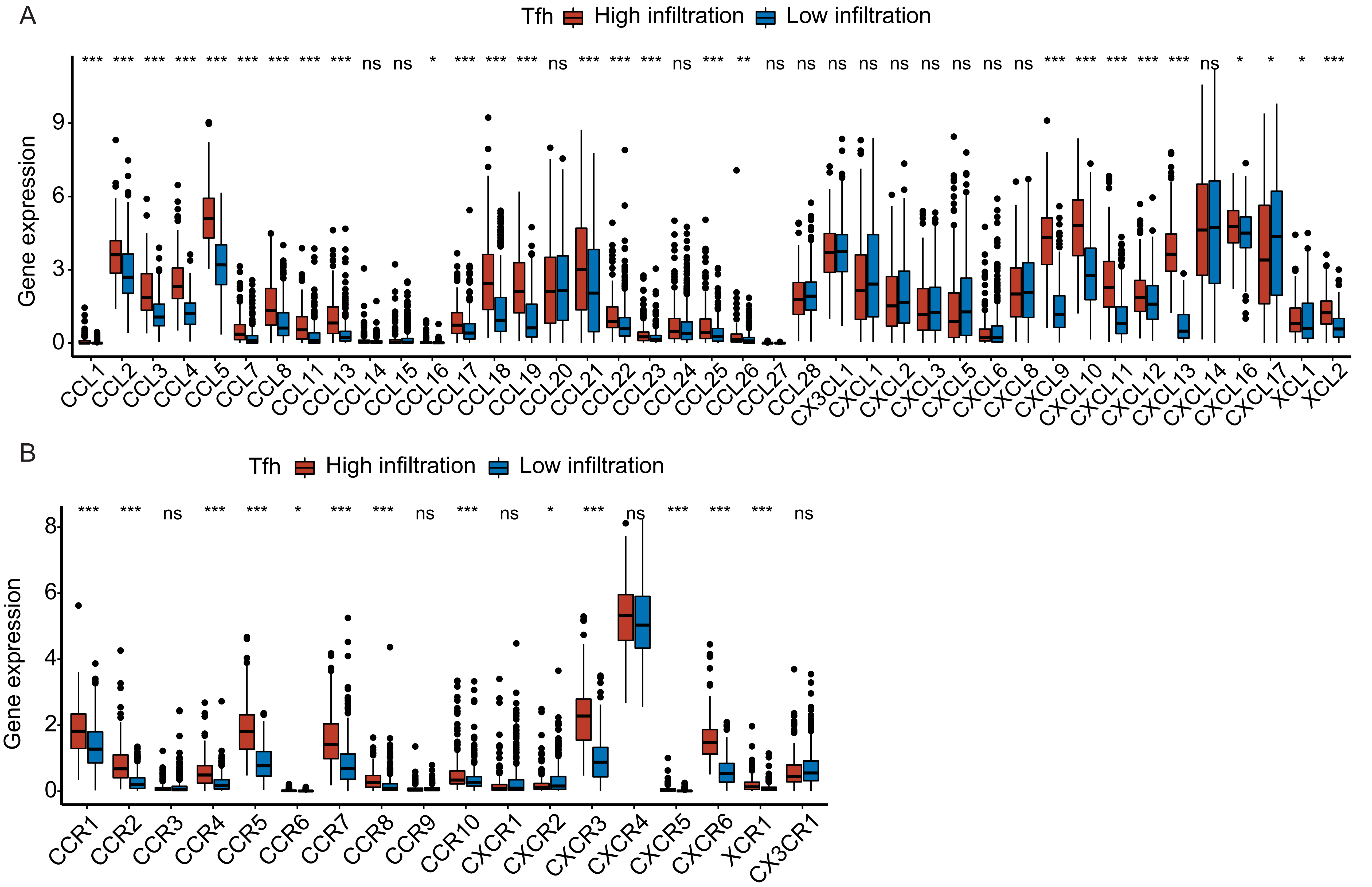
A
Tfh High infiltration Low infiltration
Gene expression
0
3
6
9
*** *** *** *** *** *** *** *** *** ns ns * *** *** *** ns *** *** *** ns *** ** ns ns ns ns ns ns ns ns ns *** *** *** *** *** ns * * * ***
CCL1 CCL2 CCL3 CCL4 CCL5 CCL7 CCL8 CCL11 CCL13 CCL14 CCL15 CCL16 CCL17 CCL18 CCL19 CCL20 CCL21 CCL22 CCL23 CCL24 CCL25 CCL26 CCL27 CCL28 CX3CL1 CXCL1 CXCL2 CXCL3 CXCL5 CXCL6 CXCL8 CXCL9 CXCL10 CXCL11 CXCL12 CXCL13 CXCL14 CXCL16 CXCL17 XCL1 XCL2
B
Tfh High infiltration Low infiltration
Gene expression
0
2
4
6
8
*** *** ns *** *** * *** *** ns *** ns * *** ns *** *** *** ns
CCR1 CCR2 CCR3 CCR4 CCR5 CCR6 CCR7 CCR8 CCR9 CCR10 CXCR1 CXCR2 CXCR3 CXCR4 CXCR5 CXCR6 XCR1 CX3CR1
